# QTL mapping reveals key factors related to the isoflavone contents and agronomic traits of soybean (*Glycine max*)

**DOI:** 10.1186/s12870-023-04519-x

**Published:** 2023-10-26

**Authors:** Jung Min Kim, Ji Su Seo, Jeong Woo Lee, Jae Il Lyu, Jaihyunk Ryu, Seok Hyun Eom, Bo-Keun Ha, Soon-Jae Kwon

**Affiliations:** 1 Advanced Radiation Technology Institute, Korea Atomic Energy Research Institute, Jeongeup, 56212 Republic of Korea; 2 Department of Applied Plant Science, College of Agriculture and Life Sciences, Chonnam National University, Gwangju, 61186 Republic of Korea; 3 Department of Agricultural Biotechnology, National Institute of Agricultural Sciences, RDA, Jeonju, 54874 Republic of Korea; 4 Department of Smart Farm Science, College of Life Sciences, Kyung Hee University, Yongin, 17104 Republic of Korea

**Keywords:** Soybean, Isoflavone, Single nucleotide polymorphism (SNP), Radiation breeding, Quantitative trait locus (QTL)

## Abstract

**Background:**

Soybean is a valuable source of edible protein and oil, as well as secondary metabolites that can be used in food products, cosmetics, and medicines. However, because soybean isoflavone content is a quantitative trait influenced by polygenes and environmental interactions, its genetic basis remains unclear.

**Results:**

This study was conducted to identify causal quantitative trait loci (QTLs) associated with soybean isoflavone contents. A mutant-based F_2_ population (190 individuals) was created by crossing the Korean cultivar Hwanggeum with low isoflavone contents (1,558 µg g^−1^) and the soybean mutant DB-088 with high isoflavone contents (6,393 µg g^−1^). A linkage map (3,049 cM) with an average chromosome length of 152 cM was constructed using the 180K AXIOM® SoyaSNP array. Thirteen QTLs related to agronomic traits were mapped to chromosomes 2, 3, 11, 13, 19, and 20, whereas 29 QTLs associated with isoflavone contents were mapped to chromosomes 1, 3, 8, 11, 14, 15, and 17. Notably, the qMGLI11, qMGNI11, qADZI11, and qTI11, which located Gm11_9877690 to Gm11_9955924 interval on chromosome 11, contributed to the high isoflavone contents and explained 11.9% to 20.1% of the phenotypic variation. This QTL region included four candidate genes, encoding β-glucosidases 13, 14, 17–1, and 17–2. We observed significant differences in the expression levels of these genes at various seed developmental stages. Candidate genes within the causal QTLs were functionally characterized based on enriched GO terms and KEGG pathways, as well as the results of a co-expression network analysis. A correlation analysis indicated that certain agronomic traits (e.g., days to flowering, days to maturity, and plant height) are positively correlated with isoflavone content.

**Conclusions:**

Herein, we reported that the major QTL associated with isoflavone contents was located in the interval from Gm11_9877690 to Gm11_9955924 (78 kb) on chromosome 11. Four β-glucosidase genes were identified that may be involved in high isoflavone contents of soybean DB-088. Thus, the mutant alleles from soybean DB-088 may be useful for marker-assisted selection in developing soybean lines with high isoflavone contents and superior agronomic traits.

**Supplementary Information:**

The online version contains supplementary material available at 10.1186/s12870-023-04519-x.

## Background

Soybean contains functional compounds, such as primary (protein and oil) and secondary (tocopherol, saponin, and isoflavone) metabolites, and is an important cash crop because it is used for the production of food and feed, cosmetics, and biofuels [1]. Isoflavones, which are produced exclusively by species in the family Fabaceae (e.g., soybean), contribute to plant defense systems by serving as precursors of glyceollins and major phytoalexins as well as signaling molecules mediating plant–microbe interactions in the rhizosphere [2, 3]. Most isoflavones (80%–90%) are localized in cotyledons (700–5,300 µg g^−1^), among which malonyl-glucoside isoforms account for the largest proportion (60%–80%) [4–8]. The following 12 isoflavone forms have been identified in soybean seeds: daidzein, glycitein, genistein, daidzin, glycitin, genistin, 6-O-malonyl daidzin, 6-O-malonyl glycinin, 6-O-malonyl genistin, 6-O-acetyl daidzin, 6-O-acetyl glycinin, and 6-O-acetyl genistin [9]. Although almost all legume crops contain isoflavones, the isoflavone content in soybean is more than 100-fold greater than that in other legumes, including chickpea, faba bean, lentil, and peanut [10]. Isoflavone biosynthesis is initiated through the phenylpropanoid pathway, with the resulting compounds subsequently converted to their conjugated forms (i.e., glucosyl- and malonyl-glucosides) and then stored in vacuoles [11]. Isoflavones form a subgroup of phytoestrogens that help to prevent breast cancer, osteoporosis, heart disease, and menopausal symptoms [12]. Because of their positive effects on human health due to their antioxidant and anti-tumorigenic activities, there is increasing interest in soybean isoflavones worldwide [13].

Despite their importance, determining the genetic basis of isoflavone biosynthesis and accumulation has been difficult, with previous studies having limited success in accurately identifying the associated quantitative trait loci (QTLs). The isoflavone content is considered to be a quantitative trait primarily influenced by minor QTLs. Earlier research revealed the isoflavone content may vary by up to 3-fold depending on the effects of the genotype, environment, and genotype × environment interaction [14–16]. Linkage mapping is a typical method used to identify genomic loci related to complex qualitative/quantitative traits, with the recombination frequency of bi-parental populations (e.g., F_2_, DH, RIL, and NIL populations) used to identify causal alleles [17, 18]. Although the F_2_ generation undergoes only a single meiotic cycle, which makes linkage mapping challenging, it is useful for quickly and easily generating populations [19]. Additionally, QTL mapping using an F_2_ population is an efficient way to analyze qualitative traits, such as disease resistance, as well as quantitative traits, including metabolite contents (e.g., protein, oil, and sucrose). However, secondary metabolite contents are generally less heritable than the contents of other major compounds, but the high heritability of specific isoflavone contents has been reported [20–24]. Accordingly, QTL analyses may be applicable for these isoflavone forms in soybean seeds.

During the past decade, molecular markers have been used to construct linkage maps for QTL analyses. Many researchers have tried to reveal the genetic factors underlying complex traits using various molecular markers, including restriction fragment length polymorphisms, simple sequence repeats (SSRs), and single nucleotide polymorphisms (SNPs) [25–27]. The first linkage mapping study for isoflavones, which was conducted by Njiti et al. [28], involved the RIL mapping population of ‘Essex’ × ‘Forrest’ and 201 SSR markers. Meksem et al. [29] constructed a linkage map (2,823 cM) with an average marker spacing of 26 cM using 133 SSR markers and the RIL population used by Njiti et al. [28]. They detected major QTLs on linkage groups A1, B1, and N. Primomo et al. [30] detected 17 isoflavone-related QTLs on 11 linkage groups using 207 RILs from ‘AC756’ × ‘RCAT Angora’ and 99 SSR markers. Zeng et al. [31] constructed a linkage map (2,020 cM) using 130 RILs and 125 SSR markers from multiple environments and detected novel QTLs (QDZF_1, QGTF_1, and QTIF_1) on linkage group F in Satt144. Gutierrez-Gonzalez et al. [32] created a linkage map (2,151 cM) comprising five QTLs using 446 SNP markers and five SSR markers and 188 RILs derived from ‘Magellan’ × ‘PI 437654’.

In the past, low-density linkage maps were constructed because of insufficient polymorphic markers and technological limitations, resulting in large gaps between markers. However, recent advances in next-generation sequencing technologies, including genotyping-by-sequencing, restriction-site-associated DNA sequencing (RAD-seq), and specific locus amplified fragment sequencing (SLAF-seq), have facilitated the identification of many SNPs relevant for the high-density genotyping of crops [33–36]. On the basis of SLAF-seq and RAD-seq analyses, Li et al. [37], Pei et al. [38], and Cai et al. [39] detected novel QTLs (qIF20-2, aG8, qMD19, qMG18, qTIF19, and qIF5-1) that explained 4.2%–59.95% of the phenotypic variation (PV) and included isoflavone-related genes and transcription factor genes. The complete soybean genome sequence was successfully assembled in 2010 [40], which accelerated the development of high-throughput SNP chip platforms, including SoySNP6K Illumina Infinium BeadChip [41], SoySNP50K Illumina Infinium BeadChip [42], 180K AXIOM® SoyaSNP array [43], NAJU 355K SoySNP array [44], and SoySNP618K array [45]. Kim et al. [46] identified a novel *GmSACPD-C* allele using the RIL population derived from a high stearic acid soybean mutant, while also completing the genotyping for QTL mapping using the SoySNP6K platform and a KASP assay. Vuong et al. [47] genotyped 553 soybean plants using SoySNP50K and revealed 14 QTLs containing 60 SNPs associated with the resistance to the soybean cyst nematode through a GWAS. Lee et al. [48] genotyped 430 soybean lines from a core collection using the 180K AXIOM® SoyaSNP array and determined that 14 novel SNPs responsible for soya-saponin biosynthesis are closely related to *Glyma.07g524600* through a GWAS. Hu et al. [49] identified qPDH1 as a pod dehiscence-related QTL that includes *Glyma09g06290* (bHLH transcription factor) on chromosome 16 using 211 soybean accessions and the NAJU 355K SoySNP array. Li et al. [48] performed a GWAS and combined the results with QTL mapping data and identified candidate genes (*E2*, *GmPRR3b*, and *GmVIP5*) associated with the flowering time and the circadian clock in 265 soybean landraces using SoySNP618K.

Mutation breeding is an effective strategy for developing new cultivars with ideal agronomic traits and functional compounds. Gamma-rays are one of the most commonly used physical mutagens (60%) for mutation breeding [50]. More than 3,402 mutant varieties of 210 species are registered in the International Atomic Energy Agency/Mutant Variety Database (http://www.iaea.org/). We previously created a mutant diversity pool (MDP) comprising 208 mutant soybean lines from seven wild-type cultivars that were irradiated with ^60^Co gamma rays (250 Gy). We subsequently performed target region amplification polymorphism (TRAP) and transposable element (TE)-TRAP marker analyses to clarify genetic associations as well as gene expression and GWAS analyses of agronomic traits and phytochemical contents using our MDP and selected a soybean mutant with high isoflavone contents (average of 7,351 μg g^−1^ over a 3-year period) [51–53]. Because there has been relatively little breeding for isoflavone contents, a QTL analysis was conducted using only a few varieties (e.g., Angora, Luheidou2, Wayao, and Zhongdou 27) with isoflavone contents of 2,246–3,791 μg g^−1^ as the parental plants [30, 37, 39, 54].

The objective of this study was to address the above-mentioned problems by (1) narrowing the large confidence interval of QTLs underlying isoflavone contents; (2) eliminating the genetic bottleneck by using a mutant-based mapping population; and (3) increasing the low isoflavone contents of the Korean soybean cultivar Hwanggeum, which has superior agronomic traits. An F_2_ mapping population consisting of 190 plants was constructed by crossing the paternal parent DB-088 with the maternal parent Hwanggeum. To reveal the genetic basis of the ideal agronomic traits and isoflavone contents, the mutant-based F_2_ population was phenotyped and genotyped using the 180K AXIOM® SoyaSNP array for the fine-mapping and QTL analyses.

## Methods

### Mapping population

The soybean mutant DB-088, which has high isoflavone contents, was selected from the MDP consisting of 208 soybean mutants at the Radiation Breeding Farm of the Korea Atomic Energy Research Institute (KAERI; 35.5699°N 126.9772°E, Jeongeup, Jeollabuk, Republic of Korea) [52]. Briefly, 1,000 seeds were collected from seven soybean cultivars and irradiated with ^60^Co gamma rays (250 Gy) at KAERI in 2008. We subsequently propagated 1,695 mutants from the M_1_ generation to the M_5_ generation via single seed descent. In the M_5_ generation, we selected mutants with distinct morphological characteristics and agronomic traits (e.g., high yield, growth type, and climate adaptability). We then constructed a mutant population consisting of 208 genotypes without redundant phenotypes and propagated it until the M_10_ generation; the population was designated as the MDP. The isoflavone contents were measured in the M_10_ generation as well as in the M_11_ and M_12_ generations to determine the year-to-year variation over a 2-year period. Finally, DB-088 was selected following the gamma irradiation of Danbaek [55].

To construct the mutant-based mapping population, F_1_ plants were generated by crossing DB-088 with Hwanggeum in the soybean breeding field of Chonnam National University (36°17′N, 126°39′E, Gwangju, Republic of Korea). A total of 190 F_2_ plants were grown in plastic pots (15 cm diameter) filled with commercial soil (Hungnong, Pyeongtaek, Republic of Korea) at the Radiation Breeding Research Farm of KAERI for the production and harvest of mature seeds.

### Examination of agronomic traits and isoflavone contents

The agronomic traits of the 190 F_2_ plants in the mapping population were evaluated at the Radiation Breeding Research Greenhouse of KAERI. More specifically, the following 13 agronomic traits were characterized according to the Korea Seeds & Variety Service standard guidelines (http://www.seed.go.kr/seed/192/subview.do): growth habit (GH; D: determinate, SD: semi-determinate, and ID: indeterminate), plant type (PT; SU: semi-upright, M: medium, and H: horizontal), leaflet shape (LS; O: ovoid and HS: heart-shaped), pod color (PC; BR: brown, DBR: dark brown, and B: black), seed color (SC; YL: yellow and GY: greenish yellow), hilum color (HC; YL: yellow and DBR: dark brown), days to flowering (DF), days to maturity (DM), plant height (PH; cm), leaf width (LW; cm), leaf length (LL; cm), number of nodes (NN), and hundred-seed weight (HSW; g).

The contents of each isoflavone and the total isoflavone (TI) content were quantified as previously described [56]. Ten randomly selected seeds were collected for each soybean plant to measure the contents of the following twelve isoflavone forms: daidzein (DZE), glycitein (GLE), genistein (GNE), daidzin (DZI), glycitin (GLI), genistin (GNI), malonyl daidzin (MDZI), malonyl glycitin (MGLI), malonyl genistin (MGNI), acetyl daidzin (ADGI), acetyl glycitin (AGLI), and acetyl genistin (AGNI). Briefly, the soybean seeds were ground to a powder, after which 1 ml 80% (v/v) methanol was added to 20 mg ground material for the 24-h extraction at room temperature in a shaking incubator (150 rpm). The extracts were filtered using 0.22 µm PTFE syringe filters prior to the ultra-performance liquid chromatography (UPLC) analysis. Twelve isoflavone standards were purchased from LC Laboratories (Woburn, MA, USA) and Nacalai Tesque (Kyoto, Japan) to identify and quantify the extracted isoflavones.

### Descriptive statistics and visualization of phenotypic data

Descriptive statistics including minimum, maximum, mean, standard deviation (SD), skewness, kurtosis, and coefficient of variation were calculated using Microsoft EXCEL 2016. The Pearson’s correlation coefficient (PCC) and analysis of variance (ANOVA) were estimated in R as follows: corr_coef (data, verbose = TRUE) function and aov (formula, data = NULL, projections = FASLE, qr = TRUE, contrasts = NULL) function in METAN package, respectively. The broad-sense heritability (*H*^*2*^) was calculated through the variability package as follows: *H*^*2*^ = *V*_*g*_/*V*_*p*_, where *V*_*g*_ is the genotypic variance and *V*_*p*_ is the phenotypic variance. A histogram for the phenotypes was generated using the hist function in R.

### DNA extraction and SNP genotyping

Fully expanded trifoliate leaves from 190 F_2_ plants and two parental plants were placed in 2 ml tubes and frozen immediately using liquid nitrogen. The leaves were ground to a fine powder using TissueLyser II (Qiagen, Valencia, CA, USA). Genomic DNA was extracted from 100 mg ground material using the DNeasy Plant Mini Kit (Qiagen). The genomic DNA quality and concentration were determined using the NanoDrop ND-1000 spectrophotometer (Thermo Fisher Scientific, Waltham, MA, USA). In addition, 190 F_2_ plants and two parental plants (Hwanggeum and DB-088) were genotyped using the 180K AXIOM® SoyaSNP array (180,961 SNP markers) according to the manufacturer instructions at DNALINK Inc. (Seoul, Republic of Korea).

### Construction of a genetic linkage map and QTL mapping

A total of 26,760 SNP markers were filtered on the basis of a lack of heterozygous alleles and non-polymorphism between the parental plants. To construct the linkage map, polymorphic SNP markers were modified to the following input formats according to the JoinMap v4.1 (Kyazma, Wageningen, Netherlands) instructions: a, maternal allele; h, hetero allele; and b, paternal allele. The JoinMap v4.1 software was used to filter and identify the significant SNPs and genetic linkage according to physical positions. All SNPs that were selected had a segregation ratio that fit the expected 1:2:1 ratio as determined by the Chi-squared test. Next, the SNPs were filtered by screening for a lack of identical loci and genetic distortion according to locus similarity and the Chi-squared test, respectively, for the linkage mapping analysis. The genetic position was estimated on the basis of the recombination frequency using Kosambi’s mapping function. Using MapQTL v6.0 (Kyazma), the map file, genotype file, and phenotype file based on the maximum logarithm of the odds (LOD) scores were determined to identify the significant QTLs using 1,000 permutations with a significance level of 0.05. On the basis of the permutations, the LOD score was set as a fixed value to detect the presence of QTLs across all chromosomes. The initial interval mapping (IM) and multiple QTL mapping (MQM) methods were used for QTL mapping. Using a map file, including genotypes and genetic positions, and the genetic linkage map with LOD peaks, the SNP positions in QTLs were reorganized with MapChart v2.2. Markers were named using the following format: q (i.e., QTL) + chromosome number + trait name + order. Information regarding the QTLs was obtained from SoyBase (https://www.soybase.org/) and Phytozome (https://phytozome-next.jgi.doe.gov/).

### Gene Ontology and Kyoto Encyclopedia of Genes and Genomes enrichment and co-expression network analyses

The candidate genes within the causal QTLs were functionally annotated according to the Gene Ontology (GO) and Kyoto Encyclopedia of Genes and Genomes (KEGG) databases and a gene co-expression network analysis was performed using the ClueGO [57] and STRING [58] Cytoscape plug-ins. To detect the enriched GO terms and KEGG pathways, the strengths of the relationships between terms and pathways were determined according to chance-corrected kappa statistics and visualized using ClueGO v.2.5.9 and CluePedia v.1.5.9. The term–term associations represent clusters of enriched functions and functional diversity based on a predefined kappa score. The load marker and network specificity were adjusted for *Glycine max* (Wm82.a2.v1) and the Global option, respectively. Gene–gene interaction networks were constructed using StringApp v.2.0.1. A confidence cutoff value of 0.65 was applied to simplify networks and eliminate duplications. Information regarding the identifiers and gene descriptions were retrieved from the String database. Data were analyzed and visualized using Cytoscape 3.9.1.

### RNA extraction and quantitative real-time PCR

Immature and mature Hwanggeum and DB-088 seeds were sampled at different developmental stages (R5, R6, R6.5, R7, and R8), with three biological replicates per sample. The seeds were immediately frozen and ground to a fine powder using liquid nitrogen and a mortar and pestle. Total RNA was extracted from the ground material using the TRIzol reagent (Invitrogen, Carlsbad, CA, USA). The RNA quantity and quality were determined using a NanoDrop ND-1000 spectrophotometer (Thermo Fisher Scientific). First-strand cDNA was synthesized from the high-quality RNA using the SuperScript III First-Strand Synthesis SuperMix (Invitrogen). Gene expression levels were analyzed by quantitative real-time PCR, which was completed using the CFX96 Real-time PCR system (Bio-Rad, Hercules, CA, USA) and the iTaq Universal SYBR Green Supermix (Bio-Rad). All steps were performed as described by the manufacturer. Relative expression levels were calculated using the 2^−ΔΔCt^ method [59], with *Fbox* serving as the reference gene. The primer sets (Table S1) were designed using Primer3plus (https://www.primer3plus.com/).

## Results

### Isoflavone contents and agronomic traits of the parents and the F_2_ population

**Fig. 1:**
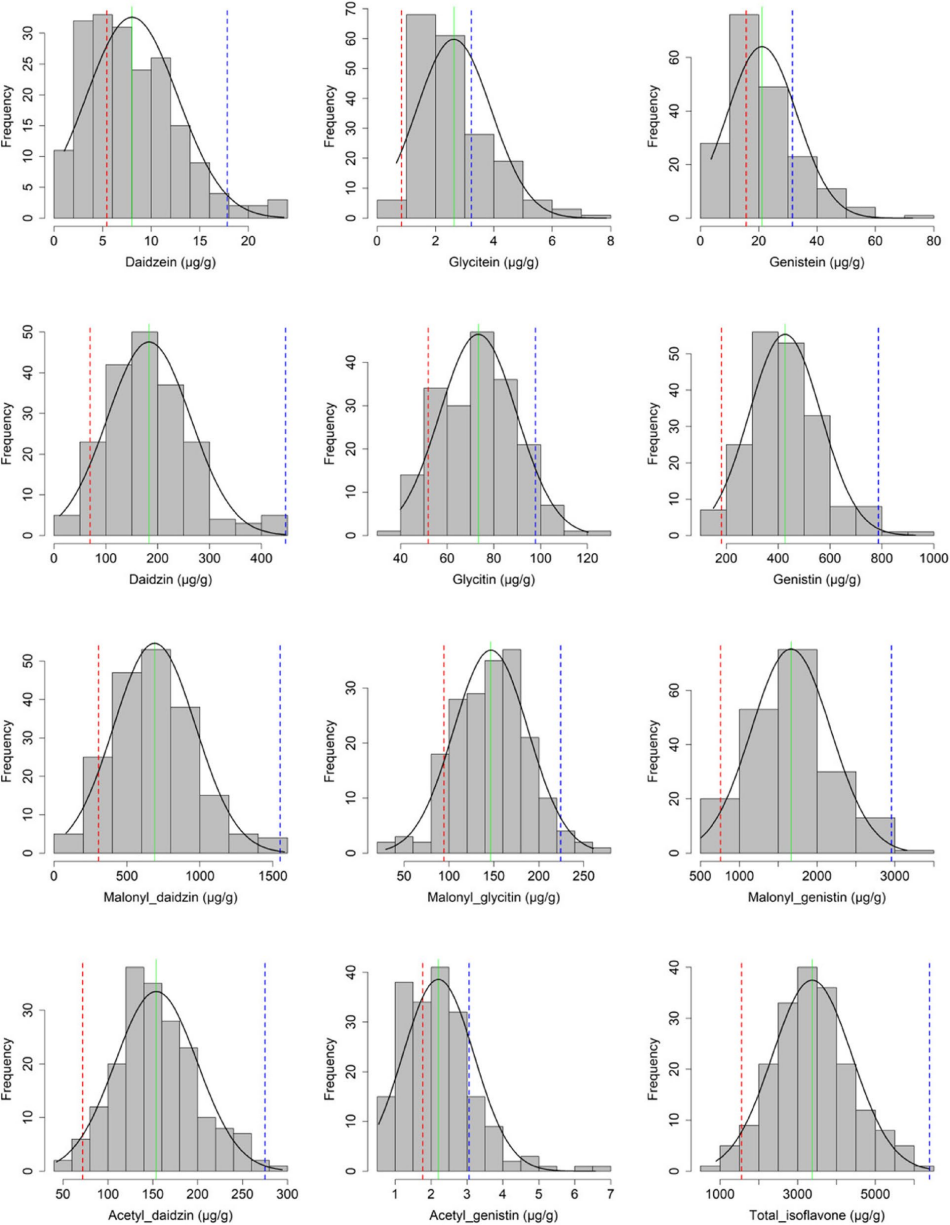
Distribution of isoflavone contents in the F_2_ population. The red and blue dotted lines indicate Hwanggeum and DB-088, respectively. The green line indicates the average for the F_2_ mapping population

The UPLC analysis revealed obvious differences between the isoflavone contents of Danbaek (original cultivar) and its mutant DB-088. The TI content was 4.04-fold higher for DB-088 (6,393 μg g^−1^) than for Danbaek (1,581 μg g^−1^). The DZI, GNI, and MGNI contents were more than 5-fold higher for DB-088 than for Danbaek, whereas there were no significant differences in the GLI and AGNI contents (Table S2). The individual isoflavone and TI contents in the F_2_ population were measured (Fig. 1). The TI contents were approximately 4.1-fold higher for DB-088 (6,393 μg g^−1^) than for Hwanggeum (1,558 μg g^−1^) (Table 1). With the exception of AGNI, the contents of each isoflavone form increased significantly in DB-088. In the F_2_ population, the isoflavone contents were as follows: aglycones [DZE (1.06–23.65 μg g^−1^), GLE (0.66–7.86 μg g^−1^), and GNE (3.65–72.78 μg g^−1^)], glucosides [DZI (11.07–430.49 μg g^−1^), GLI (39.92–120.36 μg g^−1^), and GNI (149.64–929.4 μg g^−1^)], malonyl-glucosides [MDZI (82.01–1,580.17 μg g^−1^), MGLI (29.61–260.68 μg g^−1^), and MGNI (507.4–3,154.96 μg g^−1^)], acetyl-glucosides [ADZI (42.67–294.08 μg g^−1^) and AGNI (0.54–6.58 μg g^−1^)], and TI (898.29–5,980.82 μg g^−1^). The contents of all isoflavone forms were significantly influenced by genotypic effects (Table 1). The analysis of the broad-sense heritability of isoflavone contents revealed *H*^*2*^ ranged from 0.52 (GLE) to 0.98 (GNE), indicating that genetic factors have critical effects on soybean seed isoflavone contents. Overall, the observed transgressive segregation suggested that the alleles from both parental plants contributed to the isoflavone contents in the F_2_ population.

**Table 1:** Descriptive statistics, ANOVA analysis, and broad-sense heritability of isoflavone contents in parental plants and F_2_ population

Trait^a^	Parental plants		F_2_ population				Effect	Skewness	Kurtosis	CV	*H*^*2*^
	Hwangguem	DB-088	Min	Max	Mean	SD	g				
Aglycones											
DZE	5.4 ± 0.52b^b^	17.8 ± 0.69a	1.06	23.65	7.99	4.66	***	0.89	0.81	0.59	0.91
GLE	0.8 ± 0.29b	3.2 ± 0.28a	0.66	7.86	2.63	1.28	***	1.24	1.6	0.60	0.52
GNE	15.7 ± 0.74b	31.6 ± 0.67a	3.65	72.78	21.03	11.97	***	1.21	1.83	0.56	0.98
Glycosides											
DZI	69.8 ± 5.63b	446.7 ± 20.55a	11.07	430.49	182.85	78.23	***	0.61	0.61	0.44	0.94
GLI	51.9 ± 3.46b	97.8 ± 9.24a	39.92	120.36	73.3	16.36	***	0.13	-0.41	0.25	0.70
GNI	181.7 ± 9b	786.4 ± 38.17a	149.64	929.4	425.69	135.03	***	0.60	0.66	0.33	0.93
Malonyl glycosides											
MDZI	305.4 ± 7.98b	1550.9 ± 43.21a	82.01	1580.17	688.89	273.34	***	0.40	0.27	0.40	0.97
MGLI	94.5 ± 10.72b	224.5 ± 24.8a	29.61	260.68	146.63	41.11	***	-0.12	0.18	0.30	0.82
MGNI	759.1 ± 1.98b	2956.3 ± 87.19a	507.4	3154.96	1663.75	497.4	***	0.34	0.02	0.30	0.97
Acetyl glycosides											
ADZI	71.9 ± 2.26b	275 ± 6.84a	42.67	294.08	153.77	44.62	***	0.34	0.14	0.30	0.95
AGNI	1.8 ± 1.34a	3.1 ± 0.23a	0.54	6.58	2.2	1	***	1.11	2.44	0.50	0.72
Total isoflavone	1558 ± 26.89b	6393.4 ± 189.87a	898.29	5980.82	3368.71	992.85	***	0.18	-0.04	0.30	0.96

*DZE* Daidzein, *GLE* Glycitein, *GNE* Genistein, *DZI* Daidzin, *GLI* Glycitin, *GNI* Genistin, *MDZI* Malonyl daidzin, *MGLI* Malonyl glycitin, *MGNI* Malonyl genistin, *ADZI* Acetyl daidzin, *AGLI* Acetyl glycitin, *AGNI* Ccetyl genistin, *TI* Total isoflavone, *ns* Not significant, *Min* Minimum, *Max* Maximum, *SD* Standard deviation, *g* Genotype effect, *b* Block effect, *CV* Coefficient of variation, *H*^*2*^ Broad-sense heritability

^a^ µg g^−1^

^b^ The letters adjacent to average ± standard deviation indicates the result of Fisher’s LSD test at the 5% level (*n* = 3)

^***^ Indicates significant at *p* < 0.001

In contrast to Danbaek, DB-088 is characterized by indeterminate growth, resulting in delayed flowering and maturation, which makes cultivation and breeding challenging. Therefore, we attempted to increase the isoflavone contents of Hwanggeum, which has large seeds, matures relatively early, and exhibits determinate growth. Compared with DB-088 during the growth, maturity, and harvesting stages, Hwanggeum had superior agronomic traits, including FT, DM, GH, and HSW (Table S3). Seven quantitative traits (DF, DM, PH, LW, LL, NN, and HSW) differed significantly between the parental plants (Figure S1). In the F_2_ population, 13 agronomic traits were evaluated (Figure S2), of which only HC fit the 3:1 segregation ratio (χ^2^ = 0.3439 and *P* = 0.5576).

### Correlation between the isoflavone contents and agronomic traits

**Fig. 2:**
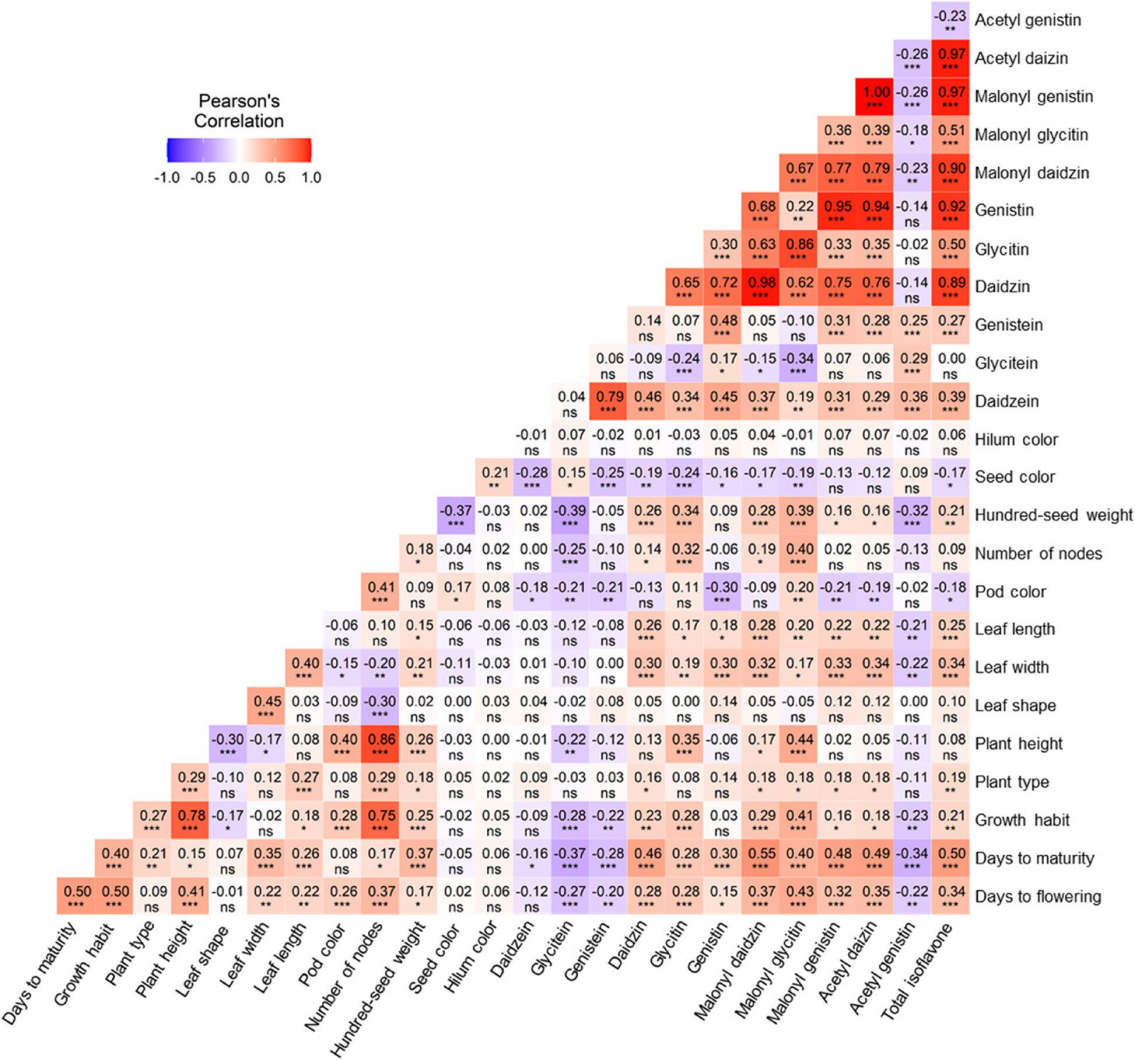
Correlations between agronomic traits and isoflavone contents. Pearson’s correlation coefficient (− 1 to + 1) indicates how strongly two variables are linearly related. *, *P* < 0.05; **, *P* < 0.01; ***, *P* < 0.001; and ns, not significant

We performed a Pearson correlation analysis to evaluate the correlations among agronomic traits and between agronomic traits and isoflavone contents in the F_2_ population (Fig. 2). Among the agronomic traits, there were strong positive correlations between PH and NN (*r* = 0.86, *P* < 0.001), GH and PH (*r* = 0.78, *P* < 0.001), GH and NN (*r* = 0.75, *P* < 0.001), DF and DM (*r* = 0.50, *P* < 0.001), and DF and GH (*r* = 0.50, *P* < 0.001), whereas there were negative correlations between PH and LS (*r* =  − 0.30, *P* < 0.001), LS and NN (*r* =  − 0.30, *P* < 0.001), and HSW and SC (*r* =  − 0.37, *P* < 0.001). The analysis of the isoflavone contents indicated the TI content was highly positively correlated with the DZI (*r* = 0.89, *P* < 0.001), MDZI (*r* = 0.90, *P* < 0.001), GNI (*r* = 0.92, *P* < 0.001), MGNI (*r* = 0.97, *P* < 0.001), and ADZI (*r* = 0.97, *P* < 0.001) contents, which was in contrast to the negative correlation between the TI content and the AGNI content (*r* =  − 0.23, *P* < 0.001). Accordingly, the DZI, GNI, MDZI, MGNI, and ADZI contents contributed substantially to the TI content. The contents of three aglycones were highly correlated with the contents of the corresponding malonyl-glucosides (*r* = 0.86–0.98, *P* < 0.001). Notably, the AGNI content was negatively correlated with the contents of the other isoflavone forms, except for aglycones. Moreover, the GNI and MGNI contents were highly positively correlated with the DZI (*r* = 0.98, *P* < 0.001) and MDZI (*r* = 1, *P* < 0.001) contents, respectively.

The analysis of the correlations between the agronomic traits and 12 isoflavone forms indicated DF and DM were positively correlated with the isoflavone contents, with the exception of the aglycone and AGNI contents. Furthermore, compared with the other agronomic traits, DF (*r* = 0.34, *P* < 0.001), DM (*r* = 0.50, *P* < 0.001), and LW (*r* = 0.34, *P* < 0.001) had stronger positive correlations with the TI content. The average TI, DF, DM, and LW values for the bottom/top 20% of the F_2_ population were 1,655.72/5,279.66 μg g^−1^, 46.4/51.95 days, 127.25/151.05 days, and 7.29/8.65 cm, respectively. The differences were significant (*P* < 0.001).

### Genetic linkage map for the F_2_ population using a high-density SNP chip array

To construct the linkage map, the raw data (180,961 SNPs) were filtered using the following criteria: no missing data and monomorphic regions in the two parental plants and the F_2_ population (Table 2 and Figure S3). Of the 26,760 SNPs (14.79%) that were retained, 5,382 (20.11%) with a segregation ratio of 1:2:1 were mapped to 20 chromosomes. The number of SNPs mapped to a chromosome ranged from 114 (chromosomes 11 and 12) to 479 (chromosome 2), with an average of 269 per chromosome. The relatively even distribution of the SNP markers among the 20 chromosomes was appropriate for constructing a linkage map. The chromosome length ranged from 87.81 cM (chromosome 4, 142 SNP markers) to 241 cM (chromosome 8, 377 SNP markers), with an average length of 152.46 cM. The total length of the linkage map was 3,049.14 cM. The average interval between adjacent markers ranged from 0.38 cM (chromosome 2) to 1.58 cM (chromosome 11), whereas the largest gap between SNP markers varied from 5.80 cM (chromosome 8) to 31.26 cM (chromosome 11). On average, 98.14% of the SNP markers on each chromosome were separated by < 5 cM [range: 92.11% (chromosome 11) to 99.74% (chromosome 3)], implying the mapped SNP markers were generally clustered together on the chromosomes.

**Table 2:** Information of linkage map in F_2_ population

Chr	No. of mapped markers	Length (cM)	Average spacing (cM)	Max gap (cM)	Gap < 5 cM
Chr 1	313	165.01	0.53	10.79	99.68%
Chr 2	479	181.68	0.38	8.58	99.58%
Chr 3	380	180.07	0.47	7.65	99.74%
Chr 4	142	87.81	0.62	9.72	97.18%
Chr 5	242	107.81	0.45	9.10	98.76%
Chr 6	178	128.06	0.72	13.14	97.19%
Chr 7	308	181.38	0.59	11.15	97.40%
Chr 8	377	241.83	0.64	5.80	99.47%
Chr 9	271	152.60	0.56	17.86	98.15%
Chr 10	195	123.94	0.64	12.51	97.44%
Chr 11	114	180.34	1.58	31.26	92.11%
Chr 12	114	107.92	0.95	17.48	96.49%
Chr 13	282	189.20	0.67	19.81	98.23%
Chr 14	250	153.13	0.61	25.31	98.40%
Chr 15	308	140.89	0.46	8.36	99.03%
Chr 16	397	176.55	0.44	11.81	99.24%
Chr 17	324	180.10	0.56	13.34	98.46%
Chr 18	319	148.46	0.47	19.03	98.75%
Chr 19	180	88.95	0.49	7.37	99.44%
Chr 20	209	133.42	0.64	8.33	98.06%
Total	5,382	3,049.14			
Mean	269.10	152.46	0.62	13.42	98.14%

No. of mapped marker, number of mapped markers

*Chr* Chromosome

### QTLs for the isoflavone contents and agronomic traits

**Fig. 3:**
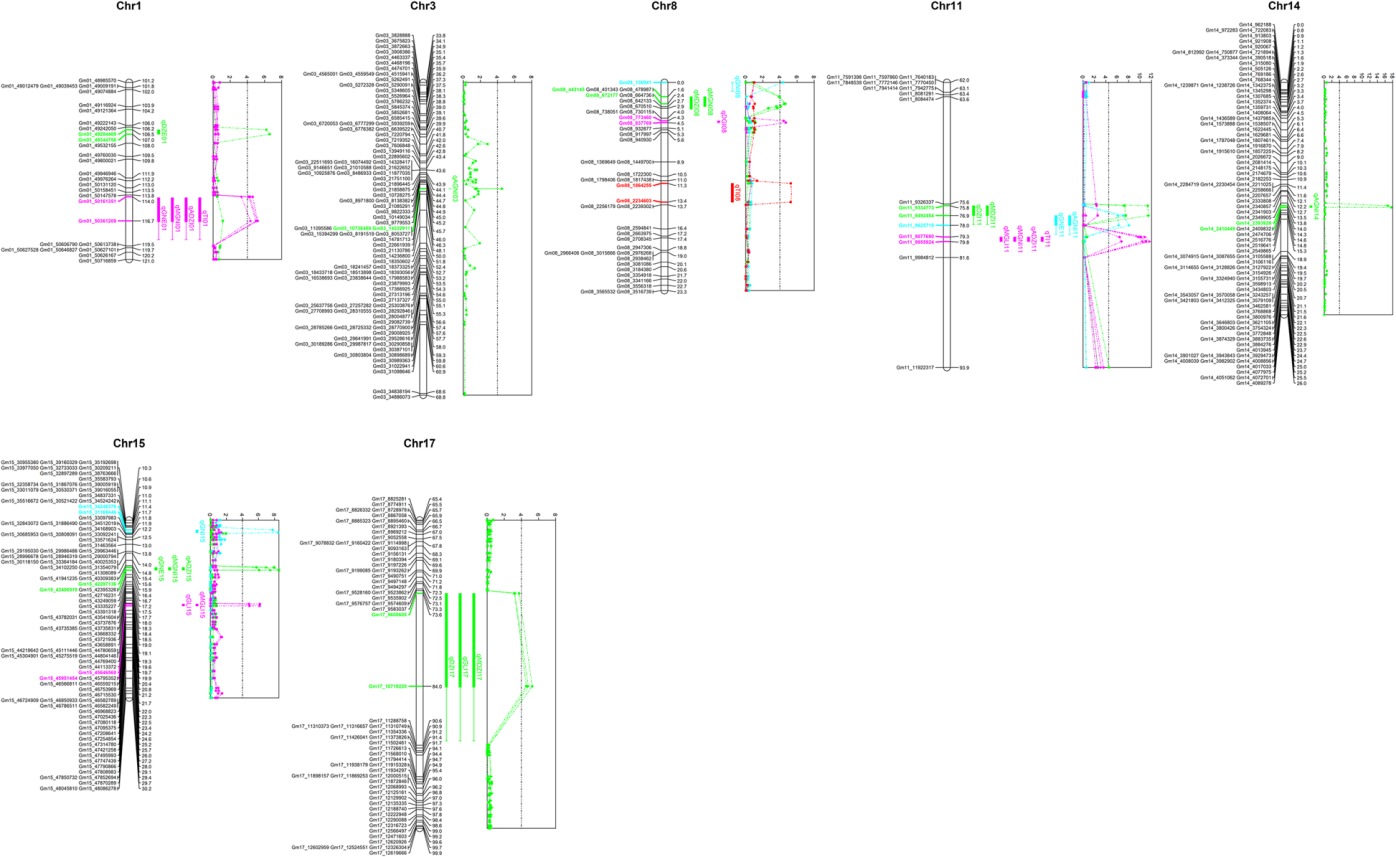
Analysis of QTLs related to isoflavone contents using a high-density linkage map of 20 chromosomes. The SNP position and genetic distance are provided on the right and left sides, respectively. The QTLs are positioned to the right of the chromosome. The QTLs in the same color are in identical positions on the chromosome

A QTL mapping analysis was also performed to reveal the causal factors associated with individual isoflavone and TI contents (Table 3 and Fig. 3). A total of 29 QTLs for the isoflavone contents (except for GLE) were mapped to chromosomes 1, 3, 8, 11, 14, 15, and 17. The QTL for DZE (qDZE01) on chromosome 1 had a LOD score of 6.61 and explained 14.8% of the PV. The three GNE-related QTLs qGNE01, qGNE11, and qGNE15 were located on chromosomes 1, 11, and 15, respectively. They had LOD scores of 5.2–8.06 and explained 8.7%–14% of the PV. The three DZI-related QTLs qDZI08, qDZI11, and qDZI17 respectively located on chromosomes 8, 11, and 17 had LOD scores of 4.68–7.48 and explained 7.8%–12.9% of the PV. The two GLI-related QTLs qGLI15 and qGLI17 were located on chromosomes 15 and 17, had LOD scores of 4.87 and 4.58, and explained 10% and 9.4% of the PV, respectively. The two GNI-related QTLs qGNI08 and qGNI15 on chromosomes 8 and 15 had LOD scores of 4.36 and 8.47 and explained 8% and 16.3% of the PV, respectively. The MDZI-related QTLs qMDZI08, qMDZI11, and qMDZI17, which were located on chromosomes 8, 11, and 17, respectively, had LOD scores of 4.56–11.46 and explained 7.2%–19.2% of the PV. The MGLI-related QTLs qMGLI11 and qMGLI15 were located on chromosomes 11 and 15, had LOD scores of 6.73 and 6.14, and explained 11.9% and 10.8% of the PV, respectively. The four MGNI-related QTLs qMGNI01, qMGNI08, qMGNI11, and qMGNI15 were located on chromosomes 1, 8, 11, and 15, respectively, had LOD scores of 4.53–11.01, and explained 6.4%–16.7% of the PV. The three QTLs (qADZI01, qADZI11, and qADZI15) for ADZI located on chromosomes 1, 11, and 15, respectively, had LOD scores of 5.01–11.7 and explained 7.9%–20.1% of the PV. The three AGNI-related QTLs qAGNI03, qAGNI11, and qAGNI14 on chromosomes 3, 11, and 14, respectively, had LOD scores of 4.51–17.65 and explained 6.4%–29.6% of the PV. The three QTLs (qTI01, qTI08, and qTI11) for the TI content on chromosomes 1, 8, and 11, respectively, had LOD scores of 4.37–10.40 and explained 7.2%–18.6% of the PV.

**Table 3:** QTL analysis for isoflavone contents in the F_2_ population

Trait	QTL	Chr	1.5-LOD interval	GD (cM)	PD (Kb)	LOD	*R*^*2*^	AE	Ref
DZE	qDZE01	1	Gm01_49284469 – Gm01_49344756	106.48–107.03	60.29	6.61	14.8	2.68	Gutierrez-Gonzalez. et al. [60]
GNE	qGNE01	1	Gm01_50161351 – Gm01_50361209	114.05–116.75	199.86	5.20	8.7	5.22	
	qGNE11	11	Gm11_9492454 – Gm11_9625710	76.88–77.95	133.26	8.06	14	6.23	
	qGNE15	15	Gm15_42297136 – Gm15_42406919	15.63–15.90	109.78	6.08	10.3	-5.54	
DZI	qDZI08	8	Gm08_773460 – Gm08_837769	4.28–4.54	64.31	4.68	7.8	-29.96	
	qDZI11	11	Gm11_9334773 – Gm11_9492454	75.82–76.88	157.68	7.48	12.9	-38.43	
	qDZI17	17	Gm17_9608605 – Gm17_10719220	73.60–84.05	1110.62	5.27	8.9	-32.47	Yoshikawa et al. [61]
GLI	qGLI15	15	Gm15_45646568 – Gm15_45951454	19.70–19.86	304.89	4.87	10	7.463	
	qGLI17	17	Gm17_9608605 – Gm17_10719220	73.60–84.05	1110.62	4.58	9.4	-6.59	Yoshikawa et al. [61]
GNI	qGNI08	8	Gm08_136941			4.36	8	-55.80	
	qGNI15	15	Gm15_31169446 – Gm15_34246376	11.36–11.66	3076.93	8.47	16.3	-81.42	Han et al. [62]
MDZI	qMDZI08	8	Gm08_443145 – Gm08_672177	1.62–2.41	229.03	4.65	7.2	-103.67	
	qMDZI11	11	Gm11_9334773 – Gm11_9492454	79.29–93.82	157.68	11.46	19.2	-164.88	
	qMDZI17	17	Gm17_9608605 – Gm17_10719220	73.60–84.05	1110.62	4.82	7.4	-103.66	Yoshikawa et al. [61]
MGLI	qMGLI11	11	Gm11_9877690 – Gm11_9955924	79.29–79.82	78.23	6.73	11.9	-20.19	
	qMGLI15	15	Gm15_45951454 – Gm15_45646568	19.70–19.86	304.89	6.14	10.8	20.25	
MGNI	qMGNI01	1	Gm01_50161351 – Gm01_50361209	114.05–116.75	199.86	5.15	7.3	190.65	
	qMGNI08	8	Gm08_443145 – Gm08_672177	1.62–2.41	229.03	4.53	6.4	-181.30	
	qMGNI11	11	Gm11_9877690 – Gm11_9955924	79.29–79.82	78.23	11.01	16.7	-286.52	
	qMGNI15	15	Gm15_42297136 – Gm15_42406919	15.63–15.90	109.78	8.54	12.6	-266.30	
ADZI	qADZI01	1	Gm01_50161351 – Gm01_50361209	114.05–116.75	199.85	5.01	7.9	17.30	
	qADZI11	11	Gm11_9877690 – Gm11_9955924	79.29–79.82	78.23	11.7	20.1	-28.08	
	qADZI15	15	Gm15_42297136 – Gm15_42406919	15.63–15.90	109.78	7.52	12.2	-22.79	
AGNI	qAGNI03	3	Gm03_10738489 – Gm03_23292786	45.73	12,554.30	4.51	6.4	-0.24	Kassem et al. [63]
	qAGNI11	11	Gm11_9492454 – Gm11_9625710	76.88–77.95	133.26	6.25	9.1	0.39	
	qAGNI14	14	Gm14_2393929 – Gm14_2410449	13.77–14.03	16.52	17.65	29.6	-0.77	
TI	qTI01	1	Gm01_50161351 – Gm01_50361209	114.05–116.75	199.86	4.37	7.2	365.35	
	qTI08	8	Gm08_1864255 – Gm08_2234603	11.26–13.41	370.35	5.26	8.8	-418.24	
	qTI11	11	Gm11_9877690 – Gm11_9955924	79.29–79.82	78.23	10.40	18.6	-613.60	

*DZE* Daidzein, *GNE* Genistein, *DZI* Daidzin, *GLI* Glycitin, *GNI* Genistin, *MDZI* Malonyl daidzin, *MGLI* Malonyl glycitin, *MGNI* Malonyl genistin, *ADZI* Acetyl daidzin, *AGNI* Acetyl genistin, *TI* Total isoflavone, *QTL* Quantitative trait locus, *Chr* Chromosome; *1.5-LOD interval* Indicates the significant location of a QTL, *Bin no.* Number of bin markers, *GD* Genetic distance, *PD* Physical distance, *Peak* Major peak in interval, *LOD* Logarithm of odds, and the ratio of the probability that a QTL is present, *R*^*2*^ The ratio (%) of the QTL explains for the phenotypic variation, *AE* The additive effect, and negative value means that it is reduced by allele of HG, *Ref* Reference

Eight intervals (Gm01_50161351–Gm01_50361209, Gm08_443145–Gm08_672177, Gm11_9334773–Gm11_9492454, Gm11_9492454–Gm11_9625710, Gm11_9877690–Gm11_9955924, Gm15_42297136–Gm15_42406919, Gm15_45646568–Gm15_45951454, and Gm17_9608605–Gm17_10719220) were detected in at least two QTLs for isoflavone contents. The intervals on chromosome 11 were associated with the negative additive effect on the TI content (− 613.6 μg g^−1^) due to the Hwanggeum allele, which explained 20.1% of the PV. The three agronomic trait-associated intervals within Gm11_9955924–Gm11_11922317 overlapped the isoflavone-related intervals (Table S4 and Figure S4). A negative additive effect reflected a decrease in the agronomic trait due to the Hwanggeum alleles [e.g., DF (− 3.01 days) and DM (− 10.48 days)]. Hence, these alleles may be useful for increasing the soybean seed isoflavone contents and improving the undesirable traits of DB-088 (e.g., late flowering, late maturity, and indeterminate growth).

### Expression patterns of the candidate genes affecting the isoflavone contents and agronomic traits

The Hwanggeum and DB-088 seed TI contents were measured at five seed developmental stages (Figure S5). The increase in the TI content varied among the examined stages. In addition, the TI content was significantly higher (*P* < 0.001) in the DB-088 seeds than in the Hwanggeum seeds at all developmental stages. Moreover, the Hwanggeum and DB-088 seed TI contents increased rapidly in the R6.5, but the TI contents in R6.5 and R7 were 3.12-fold and 3.7-fold higher in the DB-088 seeds than in the Hwanggeum seeds, respectively.

**Fig. 4:**
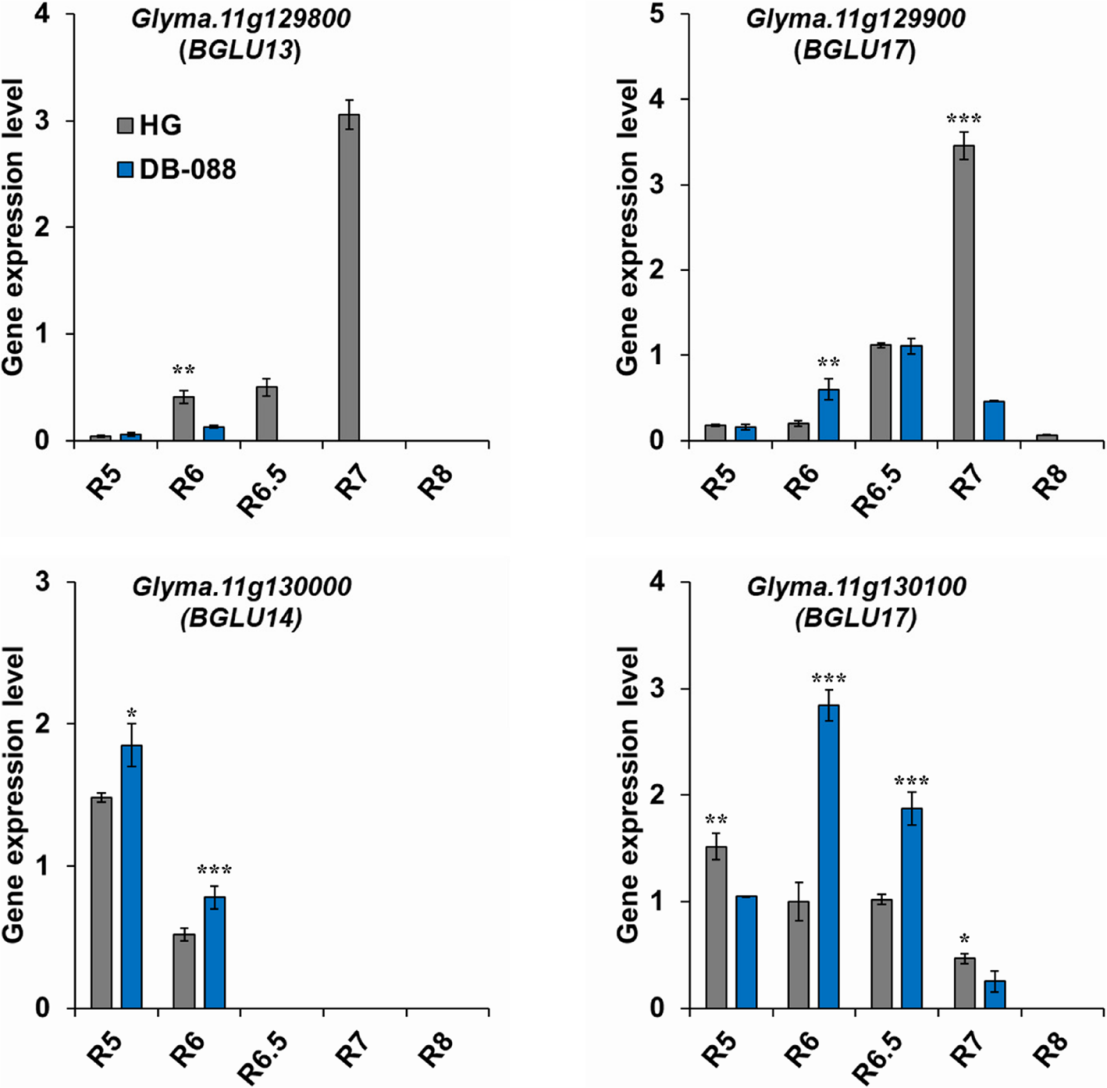
Expression levels of four candidate genes in the major interval in different seed developmental stages. The expression level was normalized to the reference gene *Fbox*. Error bars represent the standard error. *, **, and *** indicate significance at *P* < 0.05, 0.01, and 0.001, respectively

The expression levels of four candidate genes within the major interval Gm11_9877690–Gm11_9955924 were analyzed in different seed developmental stages (Fig. 4). Among the four *BGLU* (β-glucosidase) genes, the *BGLU13* (*Glyma.11g129800*) and *BGLU17-1* (*Glyma.11g129900*) expression levels in Hwanggeum increased by more than 7-fold in R7, whereas the *BGLU14* (*Glyma.11g130000*) and *BGLU17* (*Glyma.11g130100*) expression levels in DB-088 tended to be highest during the early stages (R5, R6, and R6.5). The candidate gene expression levels differed significantly between the parental plants. The interactions between genes with similar expression patterns, which were revealed by the co-expression analysis, suggest the genes may contribute to the regulation of isoflavone contents and agronomic traits.

### Co-expression networks for the candidate genes responsible for isoflavone contents and agronomic traits

**Fig. 5:**
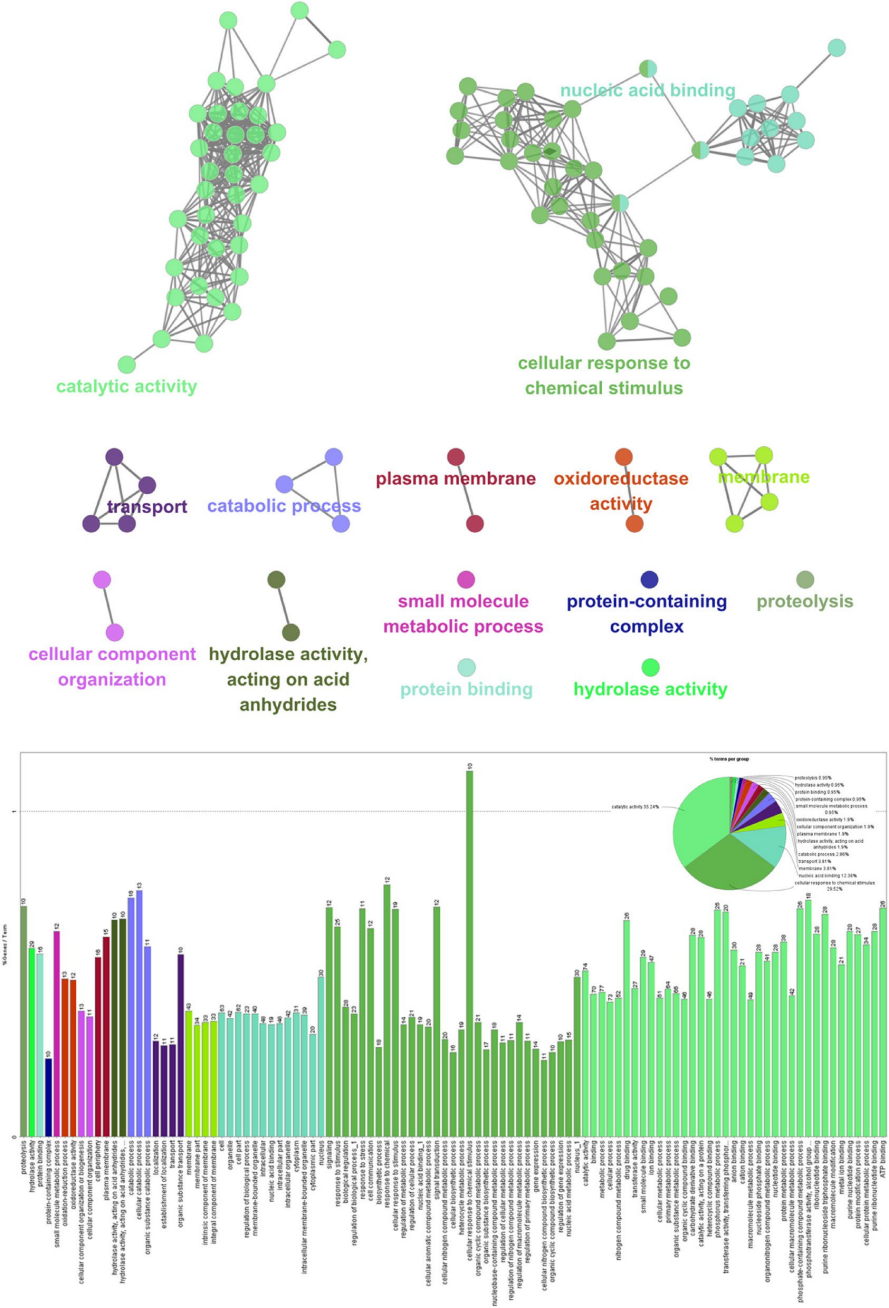
Enriched GO terms and KEGG pathways among the candidate genes associated with isoflavone contents. Nodes indicate a specific term and are linked on the basis of term–term similarity. Different colors reflect the degree of enrichment, with only the most significant term presented. The bar and node colors match the color of the corresponding term. The node size reflects significance and group colors were arbitrarily selected. The percentage of genes per term and terms per group are indicated in the bar graph and pie chart, respectively. This figure was adapted with permission from the copyright holder (Kyoto Encyclopedia of Genes and Genomes)

The 267 candidate genes within the QTLs related to isoflavone contents were annotated with 102 GO terms, resulting in 298 connected gene nodes and 596 edges. These genes were categorized into 58 biological processes, 19 cellular components, and 28 molecular functions (Fig. 5). A total of 128 functionally characterized genes were annotated with 15 GO terms, including catalytic activity, nucleic acid binding, and cellular response to chemical stimulus. Many genes were annotated with multiple GO terms. The 174 candidate genes within the QTLs related to agronomic traits were used to construct the co-expression networks on the basis of gene–gene interactions. According to the enriched GO terms and KEGG pathways, the candidate genes were categorized into 49 biological processes, 17 cellular components, and 13 molecular functions (Figure S6). Detailed regarding these GO terms are provided in Tables S5 and S6.

**Fig. 6:**
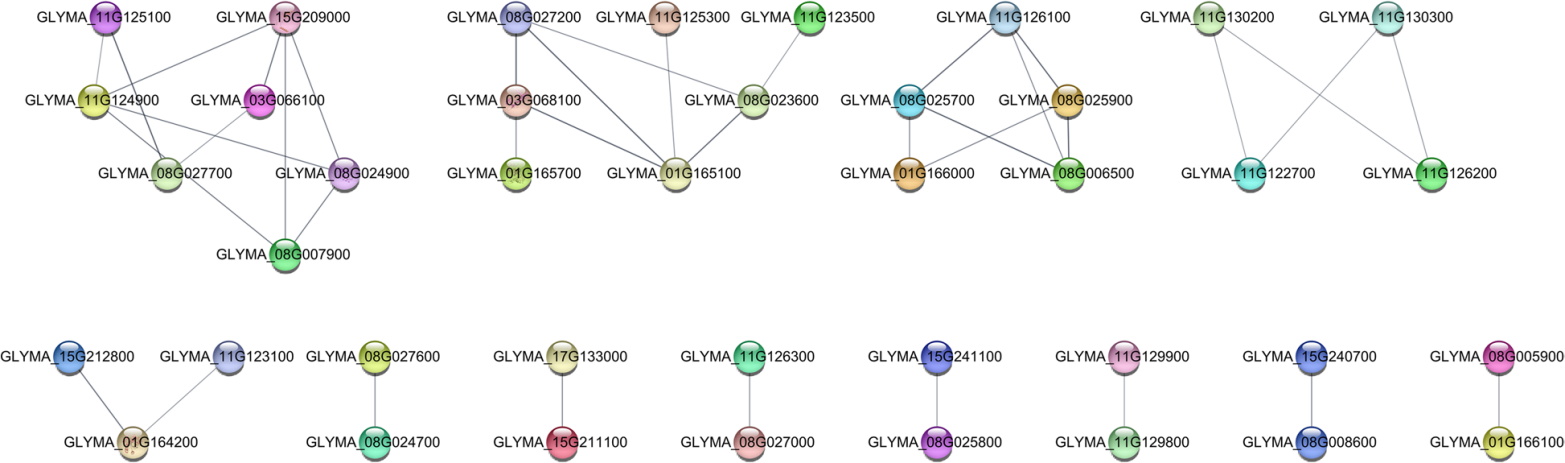
Gene–gene interaction network comprising the candidate genes associated with isoflavone contents. Edges (gray line) indicate gene–gene interactions. Node (gene) colors were arbitrarily selected. High confidence scores are indicated by thick and dark lines

The co-expression network analysis indicated 40 genes were either directly or indirectly linked through one or more interactions with the isoflavone contents, reflecting the similarity in their functions (Fig. 6). Additionally, within the causal QTLs, one gene related to agronomic traits and four genes associated with isoflavone contents were involved in gene–gene interactions. Specifically, within the major interval for agronomic traits on chromosome 11, *Glyma.11g129900* (β-glucosidase 17, *BGLU17*) interacted with four *BGLU* genes, namely *Glyma.11g129500* (*BGLU13*), *Glyma.11g129700* (*BGLU17*), *Glyma.11g129300* (*BGLU15*), and *Glyma.11g129600* (*BGLU17*), which were responsible for DF, GH, PT, and LL (Figure S7). In terms of the isoflavone contents, an interaction was detected between two *BGLU* genes, *Glyma.11g129800* (*BGLU13*) and *Glyma.11g129900* (*BGLU17*), which were associated with MGLI, MGNI, ADZI, and TI (Fig. 6).

## Discussion

Isoflavones are polyphenolic compounds that are highly abundant in soybean [10]. Soybean isoflavones are multifunctional compounds important for legume–microbe interactions and the estrogenic effects of 17B-estradiol [3, 12]. However, the practical breeding strategies for developing soybean varieties with high isoflavone contents are inefficient and inadequate. We previously developed the DB-088 mutant with high isoflavone contents via gamma irradiation [52]. Compared with the corresponding contents in Danbaek and Hwanggeum, there was an approximately 4-fold increase in the TI content (1,580.51/1,558.04 μg g^−1^, respectively) in DB-088 (aglycones: 52.61 μg g^−1^ and glucosides: 6,340.75 μg g^−1^); the contents of the aglycones and conjugated forms were 3.7-fold/2.3-fold (14.34/21.98 μg g^−1^, respectively) and 4-fold/4.1-fold (1,566.17/1,536.06 μg g^−1^, respectively) higher in DB-088, respectively (Table S2). Isoflavone conjugates, which are the main constituents in seeds, are relatively stable and soluble, making them appropriate for storage in vacuoles [64]. Additionally, isoflavone conjugates can be converted into aglycones via fermentation, thereby increasing their bioavailability for the production of pharmaceuticals as well as moisturizing and anti-aging cosmetics [65, 66].

In the current study, our objective was to identify QTLs associated with high isoflavone contents in DB-088. The SoyBase database contains relatively few QTLs related to secondary metabolites. For example, it includes 297, 81, and 31 QTLs related to the isoflavone, tocopherol, and saponin contents, respectively, whereas there are 1,032 QTLs associated with the oil content. Most earlier QTL analyses were conducted using homozygous populations, but maintaining large populations can be cumbersome, time-consuming, and costly [38, 39, 67]. This compelled us to conduct a QTL mapping analysis of isoflavone contents and agronomic traits using a heterozygous population (F_2_ generation). By identifying causal QTLs with potential candidate genes, we revealed the potential applicability of these traits. The heritability of the contents of 12 isoflavones varied, from the relatively low heritability of the aglycone contents (35%–72%) to the higher heritability of the glucoside (63%–98%), malonyl-glucoside (72%–96%), and TI (44%–99%) contents [5, 30, 68, 69]. Additionally, according to our data, the heritability of individual isoflavone contents varied from 52% (GLE) to 98% (GNE), with an average of 86.4%. The similarity in the heritability of isoflavone contents between the heterozygous and homozygous populations suggests that the isoflavone contents will be maintained at similar levels between generations. The correlation analysis showed that the TI content was highly positively correlated with the DZI, GNI, MDZI, MGNI, and ADZI contents (*r* > 0.89). Moreover, the contents of individual isoflavones and the TI content were positively correlated with the flowering time and maturity time. Azam et al. [4] also reported that MDZI (*r* = 0.92), MGNI (*r* = 0.90), DZI (*r* = 0.81), and GNI (*r* = 0.89) contents are positively correlated with the TI content, but their analysis detected a negative correlation between the ADZI and TI contents. In the present study, a delay in DF and DM by approximately 5 and 24 days, respectively, in the mapping population resulted in a 3.2-fold increase in the isoflavone content. However, aglycone and AGNI contents were negatively correlated with almost all of the examined agronomic traits, including DF [*r* =  − 0.12 (DZE) to − 0.27 (GLE)] and DM [*r* =  − 0.16 (DZE) to − 0.37(GLE)]. Our findings are consistent with those of a previous study that showed the TI content can increase by up to 60.41% depending on the maturity time [70]. Moreover, Wang et al. [71] detected a positive correlation between the isoflavone content and maturity time, with isoflavone levels increasing during the seed filling stage under cool conditions. However, the GNI (*r* =  − 0.27), DZI (*r* =  − 0.17), and GNE (*r* =  − 0.33) contents were negatively correlated with DM.

We verified 29 QTLs for isoflavone contents using a mutant-based F_2_ mapping population and 180,961 SNP markers. The QTLs mapped to chromosomes 1, 3, 8, 11, 14, 15, and 17 had LOD scores of 4.36–17.7 and explained 6.4%–29.6% of the PV, suggestive of the presence of a major QTL underlying the isoflavone contents of soybean seeds. Of these QTLs, six related to isoflavone contents were also identified in earlier studies. Major QTLs for isoflavone contents were previously localized to chromosome 5. More specifically, gen-A1, dai-A1, and tot-A1, which are related to the GNE, DZE, and TI contents, are located in a 7–13.9 cM interval between Satt236 and Sat_271 and account for 5.8%–9.2% of the PV [72], whereas qGEN5, qDAI5, and TOT5 (Satt236–Sat_271 and Satt174–Sat_236) explain 5.5%–8.3% of the PV and have a positive additive effect (50.3–161.7) [60]. Gutierrez-Gonzalez et al. [32] identified major QTLs (qGNE5, qDAI5, and qTOT5) at the same position on chromosome 5 (BARC-042999–08498–BARC-016279–02316) using a different set of RILs. Yang et al. [73] also reported that the major QTL for the DZE, GNE, and TI contents is located near Sat_217 on chromosome 5. Furthermore, Cai et al. [39] confirmed that qIF5-1 is a major isoflavone-related QTL (611.4 kb) that contains 81 candidate genes, including genes encoding transcription factors and ubiquitin-related proteins. However, in the present study, although no significant QTLs were detected on chromosome 5, eight QTLs affecting more than two types of isoflavones were mapped to chromosome 11; these QTLs explained up to 20.1% of the PV. Kassem et al. [63, 74] identified Satt197, Satt197, Satt251, and Sct26 on chromosome 11 as the major QTLs for the seed GLE content across multiple environments. These QTLs are localized to a 2.9-cM interval and explain 21%–50.2% of the PV. Additionally, qGEN11 (15.6 cM), qDAI11 (13.6 cM), and qTOT (3.7 cM) are located between BARC-04299–08498 and BARC-016279–02316 (623.4 kb) and account for 7% of the PV, whereas qGLY11 (responsible for 3% of the PV) was identified in an interval between BARC-054421–12081 and BARC-050069–09363 that includes a gene encoding 4-coumarate:CoA ligase (*4CL*) [32]. In the current study, 22 QTLs associated with more than two isoflavone forms were revealed as closely related and were located on chromosomes 1, 8, 11, 15, and 17. The QTLs in the intervals had LOD scores of 4.37–11.7 and explained 6.4%–20.1% of the PV, suggestive of the broad contributions of both parental plants and the presence of additive effects on various types of isoflavones. Some of the identified genes encode proteins involved in the phenylpropanoid pathway (e.g., flavonoid and isoflavone biosynthesis). For example, the qGLI15 and qMGLI15 QTLs appear to include a gene (*Glyma.15g240700*) encoding MAP kinase 19. Genes encoding glycosyl hydrolase family members and acyl-CoA synthetase/ligases were identified in qGNE01, qMGNI01, qMGLI11, qMGNI11, qADZI11, and qTI11. The Gm11_9877690–Gm11_9955924 interval related to the MGLI, MGNI, ADZI, and TI contents explained 11.9%–20.1% of the PV (Table 3). This interval was also related to agronomic traits, including DF, DM, GH, PT, LL, and NN. In fact, the variation in the isoflavone contents differed significantly depending on DF and DM, which is consistent with the results of our correlation analysis. Ultimately, four putative genes encoding a β-glucosidase (*BGLU*) were identified within this 78-kb interval on chromosome 11. The additive effect of this region on the TI content (613.6 μg g^−1^) was due to the mutant alleles in the paternal plant.

In Hwanggeum, *BGLU13* (*Glyma.11g129800*) and *BGLU17* (*Glyma.11g129900*) were expressed after R6.5, which marks the onset of significant isoflavone synthesis. This suggests the enhanced hydrolysis of glucosides (deglycosylation) leads to decreased isoflavone accumulation in seeds [Hwanggeum (1,711 μg g^−1^)/DB-088 (6,462 μg g^−1^)] (Fig. 4 and Figure S5). In contrast, *BGLU14* (*Glyma.11g130000*) and *BGLU17-2* (*Glyma.11g130100*) tended to be highly expressed in the early seed developmental stages in DB-088, suggesting the encoded enzymes may function as glycosyltransferases (glycosylation) involved in synthesizing isoflavone conjugates. They are also likely related to the early flowering (by 10 days) and early maturation (by 20 days) of Hwanggeum. The positive correlation between specific traits (DF and DM) and the TI content (0.34 and 0.50, respectively) suggests the traits are associated with isoflavone accumulation. The co-expression analysis indicated that the gene–gene interaction was consistent with *BGLU* expression patterns (*Glyma.11g129800* and *Glyma.11g129900*) (Fig. 4). Specifically, *BGLU17* (*Glyma.11g129900*) was associated with agronomic traits (DF, PT, GH, and LL) and isoflavone contents (MGLI, MGNI, ADZI, and TI). These findings suggest that these genes may have similar regulatory effects on isoflavone contents and agronomic traits.

To date, there has been insufficient research on the effects of *BGLU* genes on isoflavone accumulation in soybean seeds. The *BGLU13* and *BGLU14* amino acid sequences are respectively 98% and 87% similar to that of *BGLU17*, which belongs to a group of hydrolases that specifically target flavonoid and isoflavonoid conjugates [75, 76]. Previous studies characterized a β-glucosidase (β-D-glucoside glucohydrolase; EC 3.2.1.21) as a glycosyl hydrolase contributing to the plant defense system (e.g., against biotic and abiotic stresses and herbivores), lignification, and cell wall remodeling. This enzyme can catalyze the hydrolysis of glucosides to their aglycones in two steps (glycosylation and deglycosylation), resulting in structural and functional diversity [77]. It cleaves the β-glucosidic linkages of glucose conjugates in microorganisms, animals, and plants [78, 79]. A total of 47, 51, and 64 *BGLU* genes have been identified in *Arabidopsis thaliana*, *Medicago truncatula*, and *Brassica rapa*, respectively [76, 77, 80]. Ishihara et al. [81] determined that *BGLU6* is an acyl-glucose-dependent glucosyltransferase homolog that is co-expressed with phenylpropanoid biosynthetic genes; the mutation of this gene in the transgenic *bglu6* mutant line decreases the flavonol 3-O-gentiobioside 7-O-rhamnoside (F3GG7R) content in accordance with the lack of F3GG7R associated with Ler *BGLU6* genes. Our study findings were consistent with the results of previous studies showing that the total isoflavone content is approximately 4-fold higher in fully mature seeds (R7) than in immature seeds (R5) and green full-sized seeds (R6), with more than 80% of the TI content comprising glycosides and malonyl-glucosides. The glycosylation of isoflavones (glycosylation and malonylation) leads to increased solubility and stability, which is conducive to transport and storage in vacuoles. Thus, the overexpression of *BGLU13 (Glyma.11g129800) and BGLU17 (Glyma.11g129900)* in Hwanggeum likely inhibits the formation and accumulation of isoflavone-glucosides in the late seed developmental stage because of the deglycosylation. Conversely, these genes are expressed at extremely low levels in DB-088, which enables glucosides to accumulate in the seeds.

## Conclusion

We mapped the causal QTLs underlying agronomic traits and isoflavone contents using an F_2_ mapping population derived from the soybean mutant DB-088 and a high-density SNP chip array. The QTLs identified in this study included novel QTLs as well as previously reported QTLs. In the major interval on chromosome 11, we identified the following candidate genes associated with favorable agronomic traits and high TI contents: *BGLU13* (*Glyma.11g129800*), *BGLU14* (*Glyma.11g130000*), and *BGLU17* (*Glyma.11g129900* and *Glyma.11g130100*). The study results may be useful for future research aimed at developing molecular markers applicable for the breeding of new soybean lines with optimized isoflavone contents and agronomic performance.

### Supplementary Information

**Additional file 1: Figure S1.** Morphology of plants and their seeds. From left to right: Danbaek, DB-088, and Hwangguem. (A) Plants. (B) Seeds.

**Additional file 2: Figure S2.** Distribution of agronomic traits in the F_2_ population. Growth habit (D, determinate; SD, semi-determinate; ID, indeterminate); Plant type (SU, semi-upright; M, middle; H, horizontal); Leaf shape (O, ovoid; HS, heart-shaped); Pod color (BR, brown; DBR, dark brown; BK, black); Seed color (YL, yellow; GY, greenish yellow); Hilum color (YL, yellow; DBR, dark brown). The red and blue dotted lines indicate Hwangguem and DB-088, respectively. The green line indicates the average for the F_2_ mapping population.

**Additional file 3: Figure S3.** Construction of a high-density linkage map in 20 chromosomes. The SNP position and genetic distance are provided on the right and left sides, respectively.

**Additional file 4: Figure S4.** Analysis of QTLs related to agronomic traits using a high-density linkage map of 20 chromosomes. The SNP position and genetic distance are provided on the right and left sides, respectively. The QTLs are positioned to the right of the chromosome. The QTLs in the same color are in identical positions on the chromosome.

**Additional file 5: Figure S5.** Total isoflavone content in seeds of Hwanggeum and DB-088 according to developmental stages. *, **, and *** indicate significance at *p* < 0.05, 0.01, and 0.001, respectively.

**Additional file 6: Figure S6.** Enriched GO term and KEGG pathway analyses of candidate genes associated with agronomic traits. Nodes indicated a specific term and are functionally linked based on term-term similarity. Each different color indicates the term enrichment, and only the most significant term is shown. The colors of the bars and nodes match according to respective terms. The node size means significance and group color are arbitrary. The percentage of genes per term and terms per group are shown in bars label and pie chart, respectively. Figure adapted with copyright permission from the Kyoto Encyclopedia of Genes and Genomes.

**Additional file 7: Figure S7.** Gene-gene interactions network of candidate genes associated with agronomic traits. Edges (gray line) indicate gene-gene interactions and node (gene) colors are arbitrary. Higher confidence scores are represented as thicker and darker lines.

**Additional file 8: Table S1.** List of qRT-PCR primers.

**Additional file 9: Table S2.** Total and individual isoflavone contents in seeds of Danbaek, Hwangguem, and DB-088.

**Additional file 10: Table S3.** Descriptive statistics of agronomic traits in parental plants and F2 population.

**Additional file 11: Table S4.** QTL analysis for agronomic traits in the F2 population.

**Additional file 12: Table S5.** Information of GO terms for agronomic tratis.

**Additional file 13: Table S6.** Information of GO terms for isoflavone contents.

## Data Availability

The information of sequence data and positions of QTLs in this study can be obtained in SoyBase (https://www.soybase.org/) and Phytozome (https://phytozome-next.jgi.doe.gov/). Other data and materials generated during this study are included in this research article [and its supplementary information files]. The datasets about phenotype and genotype of the repository can be found below: https://github.com/jmkim0803/QTL-mapping.

## References

[1] Goldsmith PD. Economics of soybean production, marketing, and utilization. In: Soybeans. Elsevier; 2008. p. 117–50. 10.1016/b978-1-893997-64-6.50008-1.

[2] Lygin AV, Hill CB, Zernova OV, Crull L, Widholm JM, Hartman GL (2010). Response of soybean pathogens to glyceollin. Phytopathology.

[3] Sreevidya V, Srinivasa Rao C, Sullia S, Ladha JK, Reddy PM (2006). Metabolic engineering of rice with soybean isoflavone synthase for promoting nodulation gene expression in rhizobia. J Exp Bot.

[4] Azam M, Zhang S, Abdelghany AM, Shaibu AS, Feng Y, Li Y (2020). Seed isoflavone profiling of 1168 soybean accessions from major growing ecoregions in China. Food Res Int.

[5] Kim HS, Kang BK, Seo JH, Ha TJ, Kim HT, Shin SO (2019). Quantitative variation of total seed isoflavone and its compositions in Korean soybean cultivars (*Glycine**max* (L.) Merr.). Korean J Crop Sci.

[6] Tsukamoto C, Shimada S, Igita K, Kudou S, Kokubun M, Okubo K (1995). Factors affecting isoflavone content in soybean seeds: changes in isoflavones, saponins, and composition of fatty acids at different temperatures during seed development. J Agric Food Chem.

[7] Wang HJ, Murphy PA (1994). Isoflavone composition of American and Japanese soybeans in Iowa: effects of variety, crop year, and location. J Agric Food Chem.

[8] Yoon H, Yi J, taye Desta K, Shin MJ, Lee Y, Lee S (2021). Yearly variation of isoflavone composition and yield-related traits of 35 Korean soybean germplasm. Korean Soc Breed Sci.

[9] Kudou S, Fleury Y, Welti D, Magnolato D, Uchida T, Kitamura K (1991). Malonyl isoflavone glycosides in soybean seeds (*Glycine**max* Merrill). Agric Biol Chem.

[10] Setchell KD, Radd S (2000). Soy and other legumes:‘bean’around a long time but are they the ‘superfoods’ of the millennium and what are the safety issues for their constituent phytoestrogens?. Asia Pac J Clin Nutr.

[11] Yu O, Shi J, Hession AO, Maxwell CA, McGonigle B, Odell JT (2003). Metabolic engineering to increase isoflavone biosynthesis in soybean seed. Phytochemistry.

[12] Wei H, Bowen R, Cai Q, Barnes S, Wang Y (1995). Antioxidant and antipromotional effects of the soybean isoflavone genistein. Proc Soc Exp Biol Med.

[13] Rimbach G, De Pascual-Teresa S, Ewins B, Matsugo S, Uchida Y, Minihane A-M (2003). Antioxidant and free radical scavenging activity of isoflavone metabolites. Xenobiotica.

[14] Caldwell CR, Britz SJ, Mirecki RM (2005). Effect of temperature, elevated carbon dioxide, and drought during seed development on the isoflavone content of dwarf soybean [*Glycine**max* (L.) Merrill] grown in controlled environments. J Agric Food Chem.

[15] Eldridge AC, Kwolek WF (1983). Soybean isoflavones: effect of environment and variety on composition. J Agric Food Chem.

[16] Lozovaya VV, Lygin AV, Ulanov AV, Nelson RL, Daydé J, Widholm JM (2005). Effect of temperature and soil moisture status during seed development on soybean seed isoflavone concentration and composition. Crop Sci.

[17] Collard BC, Jahufer M, Brouwer J, Pang ECK (2005). An introduction to markers, quantitative trait loci (QTL) mapping and marker-assisted selection for crop improvement: the basic concepts. Euphytica.

[18] Young N (1996). QTL mapping and quantitative disease resistance in plants. Annu Rev Phytopathol.

[19] Singh B, Singh A. Mapping populations. In: Marker-assisted plant breeding: principles and practices. Springer; 2015. p. 125–50. 10.1007/978-81-322-2316-0_5.

[20] Chapman A, Pantalone V, Ustun A, Allen F, Landau-Ellis D, Trigiano R (2003). Quantitative trait loci for agronomic and seed quality traits in an F2 and F4: 6 soybean population. Euphytica.

[21] Chiari L, Piovesan ND, Naoe LK, José IC, Viana JMS, Moreira MA (2004). Genetic parameters relating isoflavone and protein content in soybean seeds. Euphytica.

[22] Fasoula VA, Harris DK, Boerma HR (2004). Validation and designation of quantitative trait loci for seed protein, seed oil, and seed weight from two soybean populations. Crop Sci.

[23] Maughan P, Maroof M, Buss G (2000). Identification of quantitative trait loci controlling sucrose content in soybean (*Glycine**max*). Mol Breed.

[24] Wang D, Diers B, Arelli P, Shoemaker R (2001). Loci underlying resistance to race 3 of soybean cyst nematode in *Glycine**soja* plant introduction 468916. Theor Appl Genet.

[25] Botstein D, White RL, Skolnick M, Davis RW (1980). Construction of a genetic linkage map in man using restriction fragment length polymorphisms. Amer J Hum Genet.

[26] Jeffreys AJ, Wilson V, Thein SL (1985). Hypervariable ‘minisatellite’regions in human DNA. Nature.

[27] Wang DG, Fan J-B, Siao C-J, Berno A, Young P, Sapolsky R (1998). Large-scale identification, mapping, and genotyping of single-nucleotide polymorphisms in the human genome. Sci.

[28] Njiti VN, Meksem K, Yuan J, Lightfoot DA, Banz WJ, Winters TA (1999). DNA markers associated with loci underlying seed phytoestrogen content in soybeans. J Med Food.

[29] Meksem K, Njiti V, Banz W, Iqbal M, Kassem MM, Hyten DL (2001). Genomic regions that underlie soybean seed isoflavone content. J Biomed Biotechnol.

[30] Primomo VS, Poysa V, Ablett GR, Jackson CJ, Gijzen M, Rajcan I (2005). Mapping QTL for individual and total isoflavone content in soybean seeds. Crop Sci.

[31] Zeng G, Li D, Han Y, Teng W, Wang J, Qiu L (2009). Identification of QTL underlying isoflavone contents in soybean seeds among multiple environments. Theor Appl Genet.

[32] Gutierrez-Gonzalez JJ, Vuong TD, Zhong R, Yu O, Lee JD, Shannon G (2011). Major locus and other novel additive and epistatic loci involved in modulation of isoflavone concentration in soybean seeds. Theor Appl Genet.

[33] Elshire RJ, Glaubitz JC, Sun Q, Poland JA, Kawamoto K, Buckler ES (2011). A robust, simple genotyping-by-sequencing (GBS) approach for high diversity species. PLoS ONE.

[34] Miller MR, Dunham JP, Amores A, Cresko WA, Johnson EA (2007). Rapid and cost-effective polymorphism identification and genotyping using restriction site associated DNA (RAD) markers. Genome Res.

[35] Sun X, Liu D, Zhang X, Li W, Liu H, Hong W (2013). SLAF-seq: an efficient method of large-scale de novo SNP discovery and genotyping using high-throughput sequencing. PLoS ONE.

[36] Thomson MJ (2014). High-throughput SNP genotyping to accelerate crop improvement. Plant Breed Biotech.

[37] Li B, Tian L, Zhang J, Huang L, Han F, Yan S (2014). Construction of a high-density genetic map based on large-scale markers developed by specific length amplified fragment sequencing (SLAF-seq) and its application to QTL analysis for isoflavone content in *Glycine**max*. BMC Genomics.

[38] Pei R, Zhang J, Tian L, Zhang S, Han F, Yan S (2018). Identification of novel QTL associated with soybean isoflavone content. Crop J.

[39] Cai Z, Cheng Y, Ma Z, Liu X, Ma Q, Xia Q (2018). Fine-mapping of QTLs for individual and total isoflavone content in soybean (*Glycine**max* L.) using a high-density genetic map. Theor Appl Genet.

[40] Schmutz J, Cannon SB, Schlueter J, Ma J, Mitros T, Nelson W (2010). Genome sequence of the palaeopolyploid soybean. Nature.

[41] Akond M, Liu S, Schoener L, Anderson JA, Kantartzi SK, Meksem K (2013). A SNP-based genetic linkage map of soybean using the SoySNP6K Illumina Infinium BeadChip genotyping array. Plant Genet Genom Biotech.

[42] Song Q, Hyten DL, Jia G, Quigley CV, Fickus EW, Nelson RL (2013). Development and evaluation of SoySNP50K, a high-density genotyping array for soybean. PLoS ONE.

[43] Lee YG, Jeong N, Kim JH, Lee K, Kim KH, Pirani A (2015). Development, validation and genetic analysis of a large soybean SNP genotyping array. Plant J.

[44] Wang J, Chu S, Zhang H, Zhu Y, Cheng H, Yu D (2016). Development and application of a novel genome-wide SNP array reveals domestication history in soybean. Sci Rep.

[45] Li YF, Li YH, Su SS, Reif JC, Qi ZM, Wang XB (2022). SoySNP618K array: a high-resolution single nucleotide polymorphism platform as a valuable genomic resource for soybean genetics and breeding. J Integr Plant Biol.

[46] Kim SH, Ryu J, Kim WJ, Kang R, Seo E, Kim G (2019). Identification of a new GmSACPD-C allele in high stearic acid mutant Hfa180 derived from gamma-ray irradiation. Mol Breed.

[47] Vuong T, Sonah H, Meinhardt C, Deshmukh R, Kadam S, Nelson R (2015). Genetic architecture of cyst nematode resistance revealed by genome-wide association study in soybean. BMC Genomics.

[48] Lee SB, Lee KS, Kim HY, Kim DY, Seo MS, Jeong SC (2022). The discovery of novel SNPs associated with group A soyasaponin biosynthesis from Korea soybean core collection. Genomics.

[49] Hu D, Kan G, Hu W, Li Y, Hao D, Li X (2019). Identification of loci and candidate genes responsible for pod dehiscence in soybean via genome-wide association analysis across multiple environments. Front Plant Sci.

[50] Kharkwal M, Pandey R, Pawar S. Mutation breeding for crop improvement. In: Plant breeding. Springer; 2004. p. 601–45. 10.1007/978-94-007-1040-5_26

[51] Hung NN, Kim D-G, Lyu JI, Park K-C, Kim JM, Kim J-B (2021). Detecting genetic mobility using a transposon-based marker system in gamma-ray irradiated soybean mutants. Plants.

[52] Kim D-G, Lyu JI, Lee M-K, Kim JM, Hung NN, Hong MJ (2020). Construction of soybean mutant diversity pool (MDP) lines and an analysis of their genetic relationships and associations using TRAP markers. Agronomy.

[53] Kim DG, Lyu JI, Lim YJ, Kim JM, Hung NN, Eom SH (2021). Differential gene expression associated with altered isoflavone and fatty acid contents in soybean mutant diversity pool. Plants.

[54] Wu D, Li D, Zhao X, Zhan Y, Teng W, Qiu L (2020). Identification of a candidate gene associated with isoflavone content in soybean seeds using genome-wide association and linkage mapping. Plant J.

[55] Kim S, Hong E, Kim Y, Lee S, Park K, Kim H (1996). A new high protein and good seed quality soybean variet “Danbaegkong”. RDA J Agric Sci (Upland & Industrial Crops).

[56] Kim JM, Lyu JI, Kim DG, Hung NN, Seo JS, Ahn JW, et al. Genome wide association study to detect genetic regions related to isoflavone content in a mutant soybean population derived from radiation breeding. Front Plant Sci. 2022:2987. 10.3389/fpls.2022.968466. 10.3389/fpls.2022.968466 PMC9433930 36061785

[57] Bindea G, Mlecnik B, Hackl H, Charoentong P, Tosolini M, Kirilovsky A (2009). ClueGO: a Cytoscape plug-in to decipher functionally grouped gene ontology and pathway annotation networks. Bioinformatics.

[58] Doncheva NT, Morris JH, Gorodkin J, Jensen LJ (2018). Cytoscape StringApp: network analysis and visualization of proteomics data. J Proteome Res.

[59] Livak KJ, Schmittgen TD (2001). Analysis of relative gene expression data using real-time quantitative PCR and the 2^−ΔΔCT^ method. Methods.

[60] Gutierrez-Gonzalez JJ, Wu X, Gillman JD, Lee JD, Zhong R, Yu O (2010). Intricate environment-modulated genetic networks control isoflavone accumulation in soybean seeds. BMC Plant Biol.

[61] Yoshikawa T, Okumoto Y, Ogata D, Sayama T, Teraishi M, Terai M, et al. Transgressive segregation of isoflavone contents under the control of four QTLs in a cross between distantly related soybean varieties. Breeding Science. 2010;60(3):243–54. 10.1270/jsbbs.60.243.

[62] Han Y, Teng W, Wang Y, Zhao X, Wu L, Li D, et al. Unconditional and conditional QTL underlying the genetic interrelationships between soybean seed isoflavone, and protein or oil contents. Plant Breeding. 2015;134(3):300–9. 10.1111/pbr.12259.

[63] Kassem M, Shultz J, Meksem K, Cho Y, Wood A, Iqbal M (2006). An updated ‘Essex’by ‘Forrest’linkage map and first composite interval map of QTL underlying six soybean traits. Theor Appl Genet.

[64] Farag MA, Huhman DV, Dixon RA, Sumner LW (2008). Metabolomics reveals novel pathways and differential mechanistic and elicitor-specific responses in phenylpropanoid and isoflavonoid biosynthesis in *Medicago**truncatula* cell cultures. Plant Physiol.

[65] Iovine B, Iannella ML, Gasparri F, Giannini V, Monfrecola G, Bevilacqua MA (2012). A comparative analysis of the photo-protective effects of soy isoflavones in their aglycone and glucoside forms. Int J Mol Sci.

[66] Vitale DC, Piazza C, Melilli B, Drago F, Salomone S (2013). Isoflavones: estrogenic activity, biological effect and bioavailability. Eur J Drug Metab Pharmacokinet.

[67] Knizia D, Yuan J, Bellaloui N, Vuong T, Usovsky M, Song Q (2021). The soybean high density ‘forrest’by ‘williams 82’snp-based genetic linkage map identifies QTL and candidate genes for seed isoflavone content. Plants.

[68] Morrison M, Cober E, Saleem M, McLaughlin N, Frégeau-Reid J, Ma B (2008). Changes in isoflavone concentration with 58 years of genetic improvement of short-season soybean cultivars in Canada. Crop Sci.

[69] Zhao Q, Qin J, Li X, Liu B, Liu Y, Yang Q (2022). Coordinate inheritance of seed isoflavone and protein in soybean. Agriculture.

[70] Zhang J, Ge Y, Han F, Li B, Yan S, Sun J (2014). Isoflavone content of soybean cultivars from maturity group 0 to VI grown in northern and southern China. J Am Oil Chem Soc.

[71] Wang C, Sherrard M, Pagadala S, Wixon R, Scott RA (2000). Isoflavone content among maturity group 0 to II soybeans. J Am Oil Chem Soc.

[72] Gutierrez-Gonzalez JJ, Wu X, Zhang J, Lee J-D, Ellersieck M, Shannon JG (2009). Genetic control of soybean seed isoflavone content: importance of statistical model and epistasis in complex traits. Theor Appl Genet.

[73] Yang K, Moon J-K, Jeong N, Chun H-K, Kang S-T, Back K (2011). Novel major quantitative trait loci regulating the content of isoflavone in soybean seeds. Genes Genom.

[74] Kassem MA, Meksem K, Iqbal M, Njiti V, Banz W, Winters T (2004). Definition of soybean genomic regions that control seed phytoestrogen amounts. J Biomed Biotechnol.

[75] Roepke J, Bozzo GG (2015). *Arabidopsis**thaliana* β-glucosidase BGLU15 attacks flavonol 3-O-β-glucoside-7-O-α-rhamnosides. Phytochemistry.

[76] Xu Z, Escamilla-Treviño L, Zeng L, Lalgondar M, Bevan D, Winkel B (2004). Functional genomic analysis of *Arabidopsis**thaliana* glycoside hydrolase family 1. Plant Mol Biol.

[77] Yang J, Ma L, Jiang W, Yao Y, Tang Y, Pang Y (2021). Comprehensive identification and characterization of abiotic stress and hormone responsive glycosyl hydrolase family 1 genes in *Medicago**truncatula*. Plant Physiol Biochem.

[78] Hsieh M-C, Graham TL (2001). Partial purification and characterization of a soybean β-glucosidase with high specific activity towards isoflavone conjugates. Phytochemistry.

[79] Opassiri R, Pomthong B, Onkoksoong T, Akiyama T, Esen A, Ketudat Cairns JR (2006). Analysis of rice glycosyl hydrolase family 1 and expression of Os4bglu12 β-glucosidase. BMC Plant Biol.

[80] Dong X, Jiang Y, Hur Y (2019). Genome-wide analysis of glycoside hydrolase family 1 β-glucosidase genes in *Brassica**rapa* and their potential role in pollen development. Int J Mol Sci.

[81] Ishihara H, Tohge T, Viehöver P, Fernie AR, Weisshaar B, Stracke R (2016). Natural variation in flavonol accumulation in Arabidopsis is determined by the flavonol glucosyltransferase BGLU6. J Exp Bot.

