# Supplementary material for: QTL mapping reveals key factors related to the isoflavone contents and agronomic traits of soybean (Glycine max)
Source: BMC Plant Biol. 2023 Oct 26;23:517. doi: 10.1186/s12870-023-04519-x (PMC10601131; doi:10.1186/s12870-023-04519-x)
Supplement: Supplementary file 1 — Additional file 1: Figure S1. Morphology of plants and their seeds. From left to right: Danbaek, DB-088, and Hwangguem. (A) Plants. (B) Seeds. [file 12870_2023_4519_MOESM1_ESM.pptx]

## Slide 1
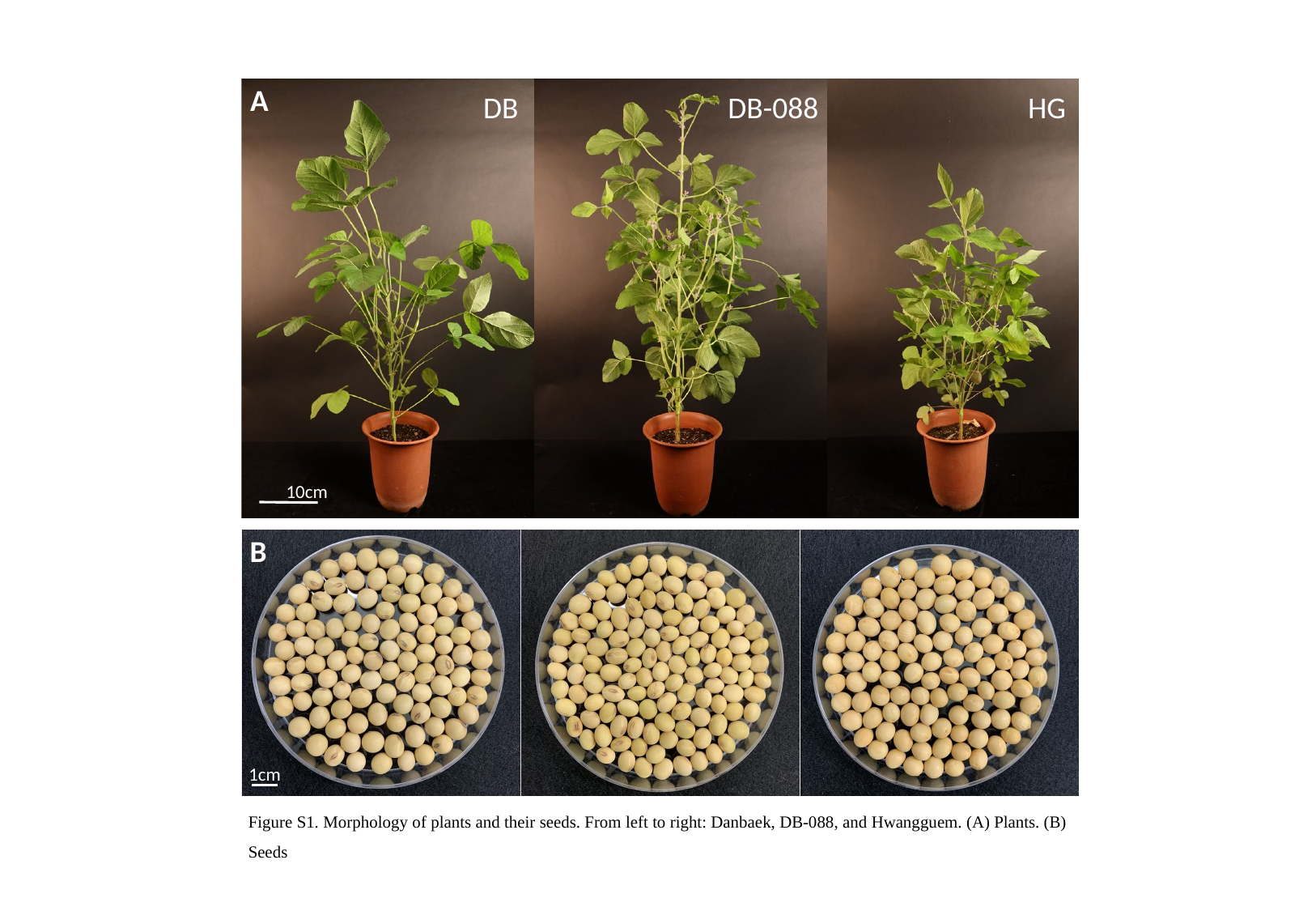

A
10cm
1cm
B
DB
DB-088
HG
Figure S1. Morphology of plants and their seeds. From left to right: Danbaek, DB-088, and Hwangguem. (A) Plants. (B) Seeds
