# Supplementary material for: QTL mapping reveals key factors related to the isoflavone contents and agronomic traits of soybean (Glycine max)
Source: BMC Plant Biol. 2023 Oct 26;23:517. doi: 10.1186/s12870-023-04519-x (PMC10601131; doi:10.1186/s12870-023-04519-x)
Supplement: Supplementary file 2 — Additional file 2: Figure S2. Distribution of agronomic traits in the F2 population. Growth habit (D, determinate; SD, semi-determinate; ID, indeterminate); Plant type (SU, semi-upright; M, middle; H, horizontal); Leaf shape (O, ovoid; HS, heart-shaped); Pod color (BR, brown; DBR, dark brown; BK, black); Seed color (YL, yellow; GY, greenish yellow); Hilum color (YL, yellow; DBR, dark brown). The red and blue dotted lines indicate Hwangguem and DB-088, respectively. The green line indicates the average for the F2 mapping population. [file 12870_2023_4519_MOESM2_ESM.pptx]

## Slide 1
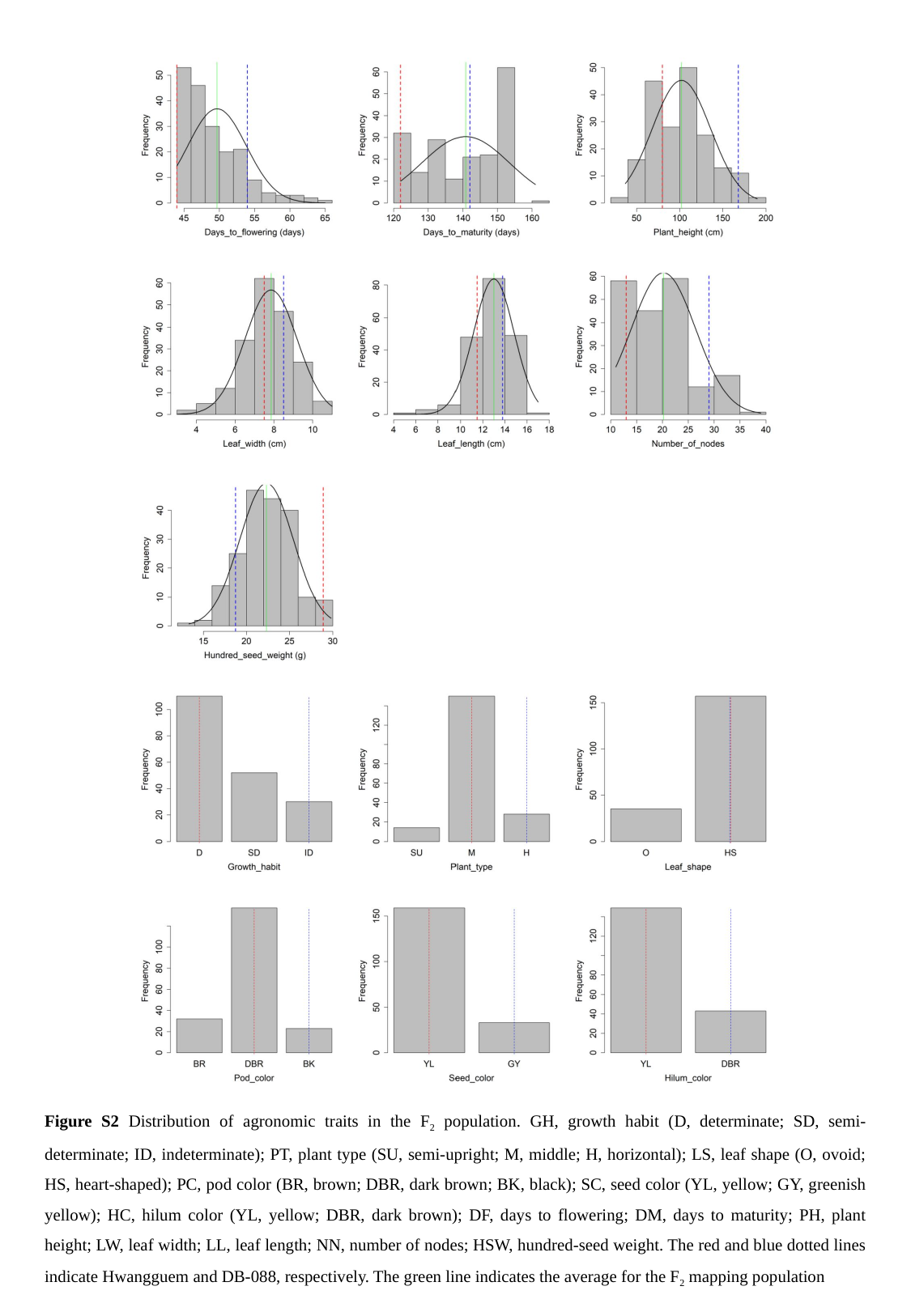

Figure S2 Distribution of agronomic traits in the F2 population. GH, growth habit (D, determinate; SD, semi-determinate; ID, indeterminate); PT, plant type (SU, semi-upright; M, middle; H, horizontal); LS, leaf shape (O, ovoid; HS, heart-shaped); PC, pod color (BR, brown; DBR, dark brown; BK, black); SC, seed color (YL, yellow; GY, greenish yellow); HC, hilum color (YL, yellow; DBR, dark brown); DF, days to flowering; DM, days to maturity; PH, plant height; LW, leaf width; LL, leaf length; NN, number of nodes; HSW, hundred-seed weight. The red and blue dotted lines indicate Hwangguem and DB-088, respectively. The green line indicates the average for the F2 mapping population
