# Supplementary material for: QTL mapping reveals key factors related to the isoflavone contents and agronomic traits of soybean (Glycine max)
Source: BMC Plant Biol. 2023 Oct 26;23:517. doi: 10.1186/s12870-023-04519-x (PMC10601131; doi:10.1186/s12870-023-04519-x)
Supplement: Supplementary file 3 — Additional file 3: Figure S3. Construction of a high-density linkage map in 20 chromosomes. The SNP position and genetic distance are provided on the right and left sides, respectively. [file 12870_2023_4519_MOESM3_ESM.pptx]

## Slide 1
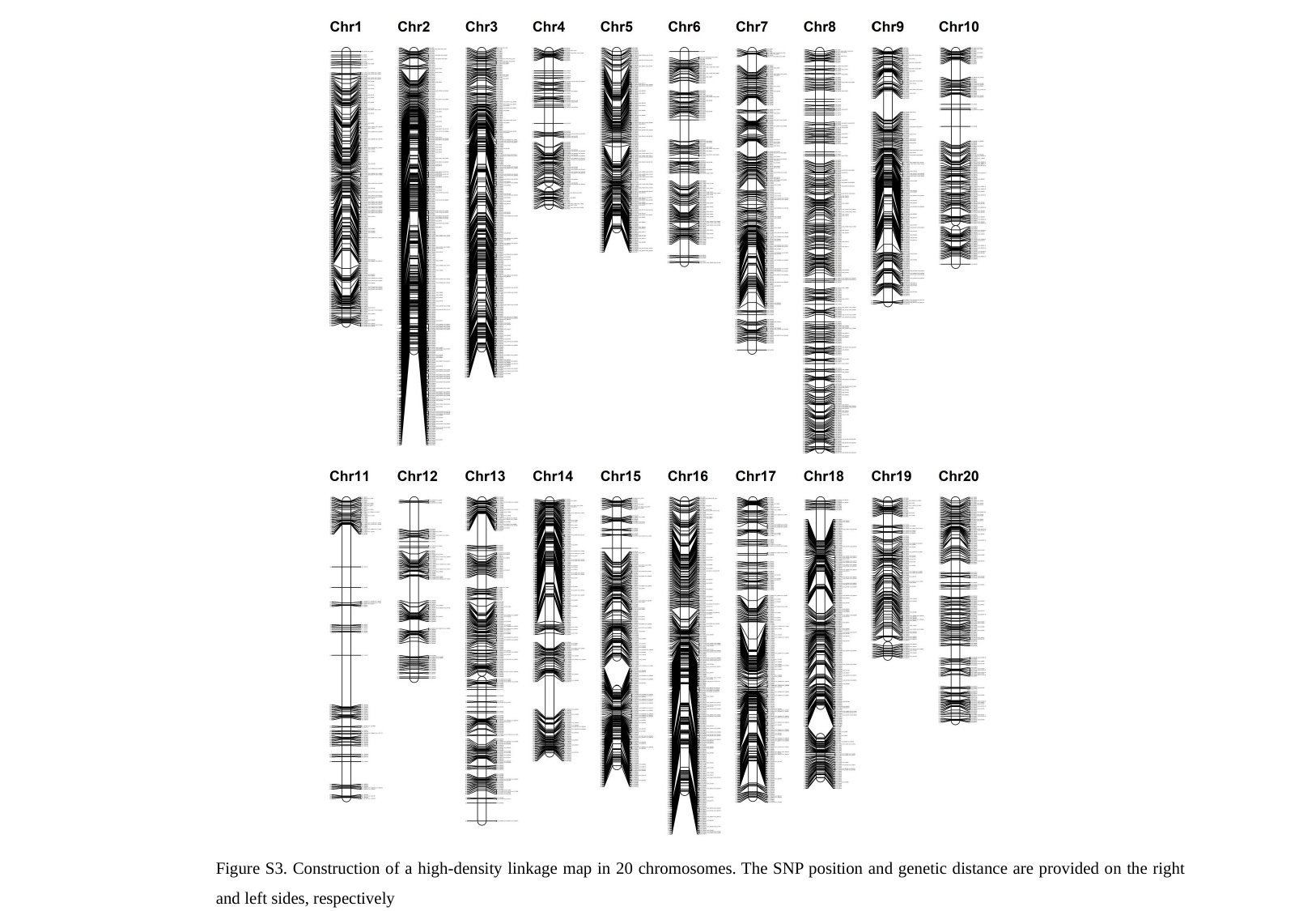

Figure S3. Construction of a high-density linkage map in 20 chromosomes. The SNP position and genetic distance are provided on the right and left sides, respectively
