# Supplementary material for: QTL mapping reveals key factors related to the isoflavone contents and agronomic traits of soybean (Glycine max)
Source: BMC Plant Biol. 2023 Oct 26;23:517. doi: 10.1186/s12870-023-04519-x (PMC10601131; doi:10.1186/s12870-023-04519-x)
Supplement: Supplementary file 4 — Additional file 4: Figure S4. Analysis of QTLs related to agronomic traits using a high-density linkage map of 20 chromosomes. The SNP position and genetic distance are provided on the right and left sides, respectively. The QTLs are positioned to the right of the chromosome. The QTLs in the same color are in identical positions on the chromosome. [file 12870_2023_4519_MOESM4_ESM.pptx]

## Slide 1
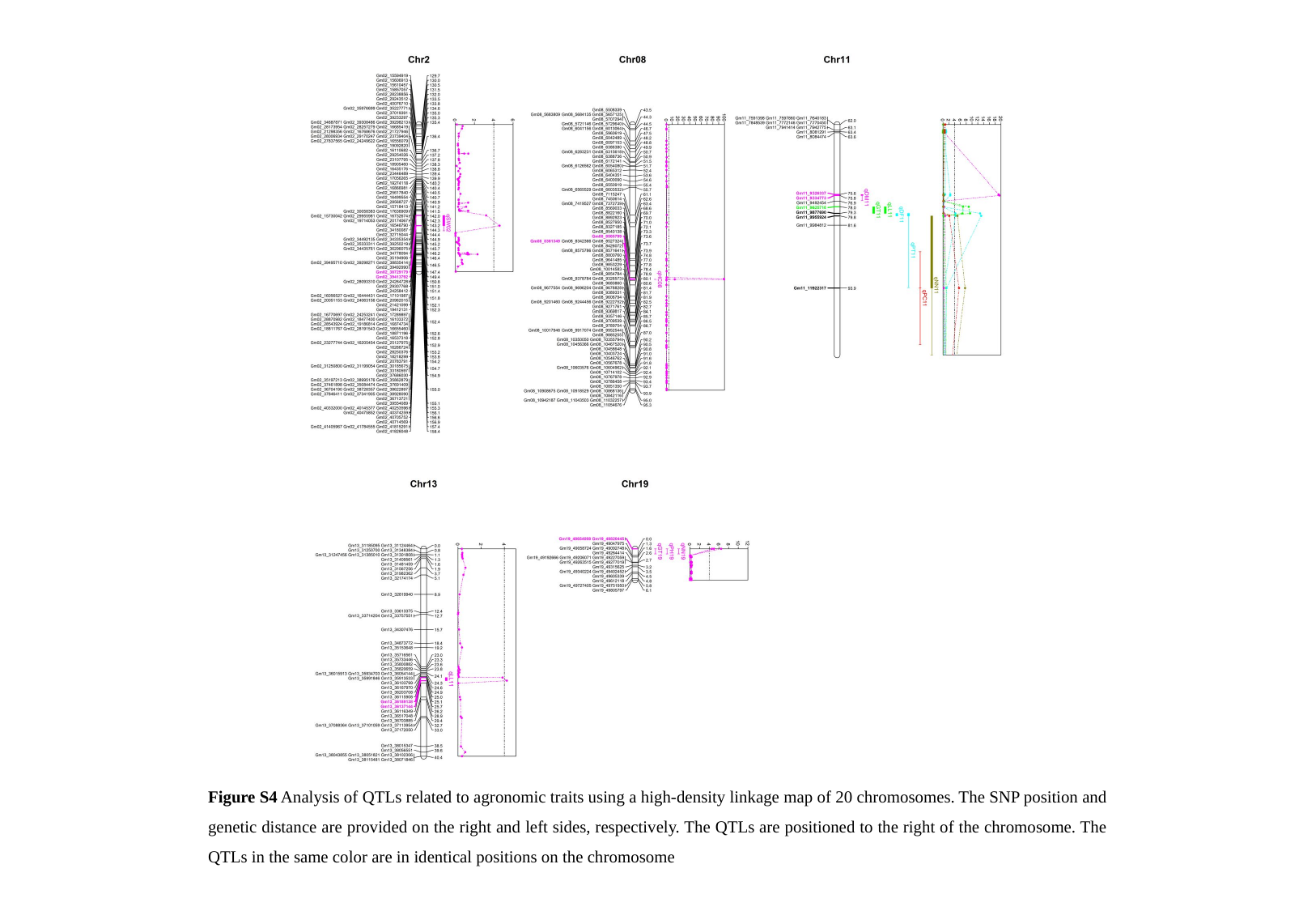

Figure S4 Analysis of QTLs related to agronomic traits using a high-density linkage map of 20 chromosomes. The SNP position and genetic distance are provided on the right and left sides, respectively. The QTLs are positioned to the right of the chromosome. The QTLs in the same color are in identical positions on the chromosome
