# Supplementary material for: QTL mapping reveals key factors related to the isoflavone contents and agronomic traits of soybean (Glycine max)
Source: BMC Plant Biol. 2023 Oct 26;23:517. doi: 10.1186/s12870-023-04519-x (PMC10601131; doi:10.1186/s12870-023-04519-x)
Supplement: Supplementary file 5 — Additional file 5: Figure S5. Total isoflavone content in seeds of Hwanggeum and DB-088 according to developmental stages. *, **, and *** indicate significance at p < 0.05, 0.01, and 0.001, respectively. [file 12870_2023_4519_MOESM5_ESM.pptx]

## Slide 1
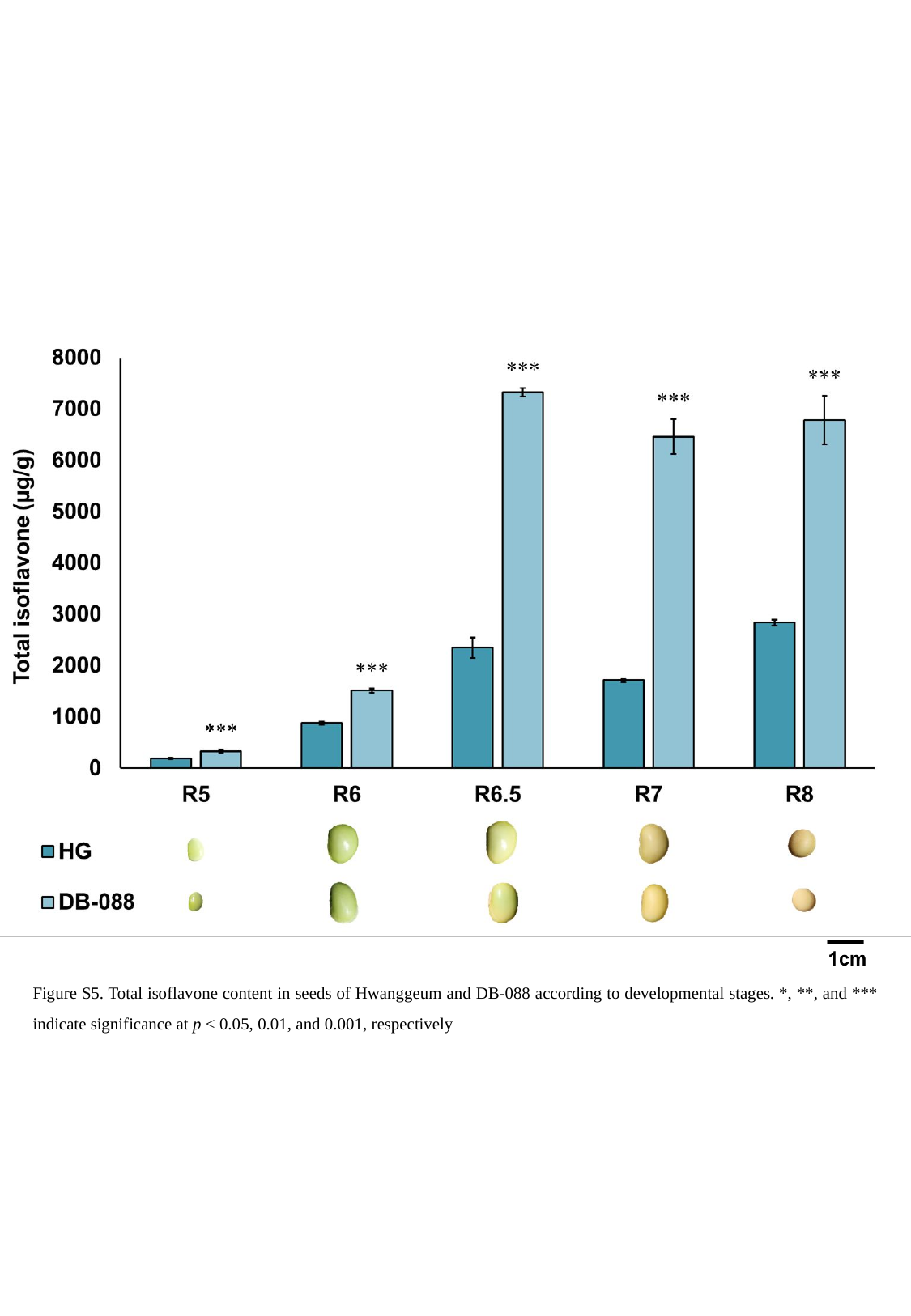

Figure S5. Total isoflavone content in seeds of Hwanggeum and DB-088 according to developmental stages. *, **, and *** indicate significance at p < 0.05, 0.01, and 0.001, respectively
