# Supplementary material for: QTL mapping reveals key factors related to the isoflavone contents and agronomic traits of soybean (Glycine max)
Source: BMC Plant Biol. 2023 Oct 26;23:517. doi: 10.1186/s12870-023-04519-x (PMC10601131; doi:10.1186/s12870-023-04519-x)
Supplement: Supplementary file 6 — Additional file 6: Figure S6. Enriched GO term and KEGG pathway analyses of candidate genes associated with agronomic traits. Nodes indicated a specific term and are functionally linked based on term-term similarity. Each different color indicates the term enrichment, and only the most significant term is shown. The colors of the bars and nodes match according to respective terms. The node size means significance and group color are arbitrary. The percentage of genes per term and terms per group are shown in bars label and pie chart, respectively. Figure adapted with copyright permission from the Kyoto Encyclopedia of Genes and Genomes. [file 12870_2023_4519_MOESM6_ESM.pptx]

## Slide 1
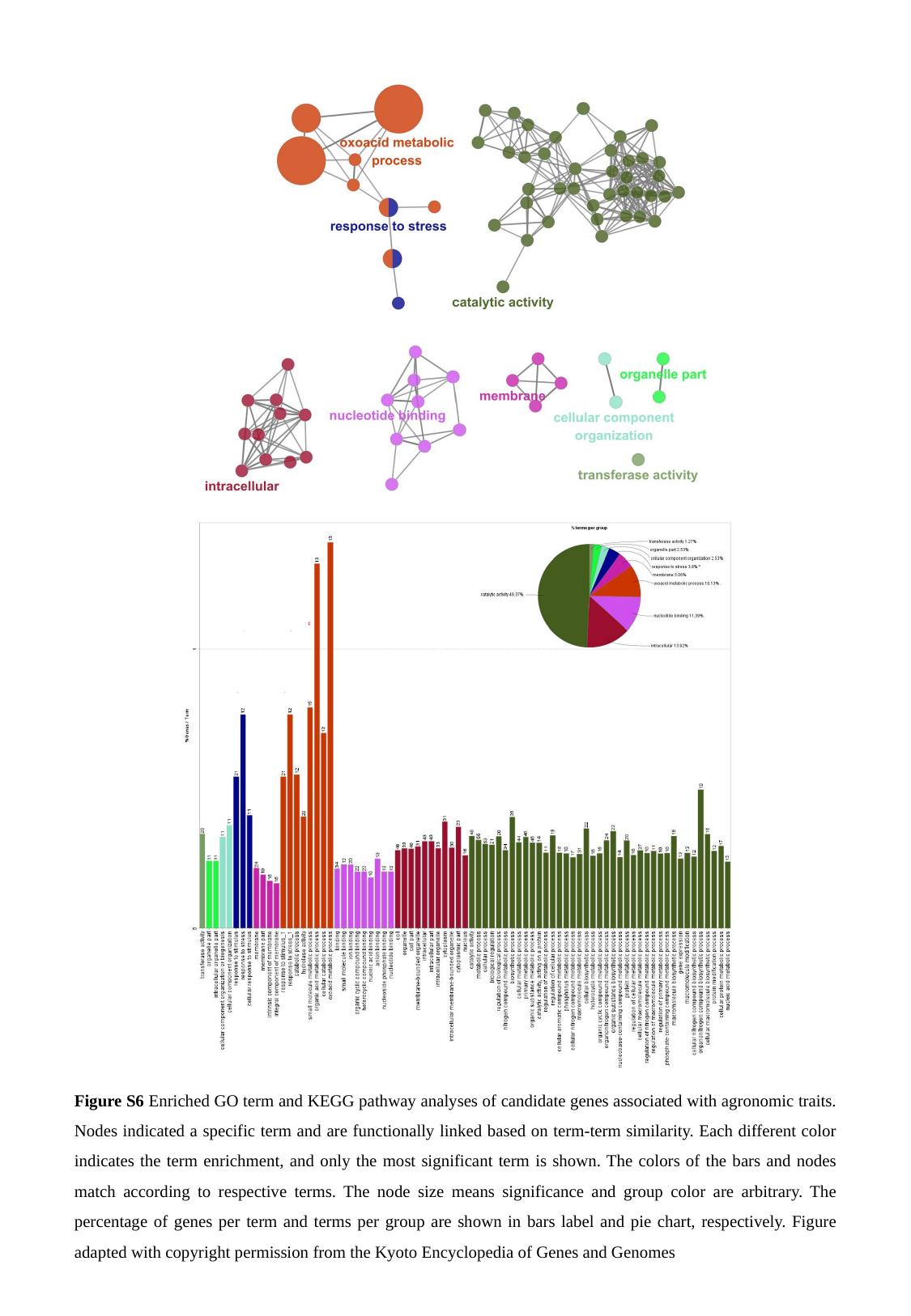

Figure S6 Enriched GO term and KEGG pathway analyses of candidate genes associated with agronomic traits. Nodes indicated a specific term and are functionally linked based on term-term similarity. Each different color indicates the term enrichment, and only the most significant term is shown. The colors of the bars and nodes match according to respective terms. The node size means significance and group color are arbitrary. The percentage of genes per term and terms per group are shown in bars label and pie chart, respectively. Figure adapted with copyright permission from the Kyoto Encyclopedia of Genes and Genomes
