# Supplementary material for: QTL mapping reveals key factors related to the isoflavone contents and agronomic traits of soybean (Glycine max)
Source: BMC Plant Biol. 2023 Oct 26;23:517. doi: 10.1186/s12870-023-04519-x (PMC10601131; doi:10.1186/s12870-023-04519-x)
Supplement: Supplementary file 7 — Additional file 7: Figure S7. Gene-gene interactions network of candidate genes associated with agronomic traits. Edges (gray line) indicate gene-gene interactions and node (gene) colors are arbitrary. Higher confidence scores are represented as thicker and darker lines. [file 12870_2023_4519_MOESM7_ESM.pptx]

## Slide 1
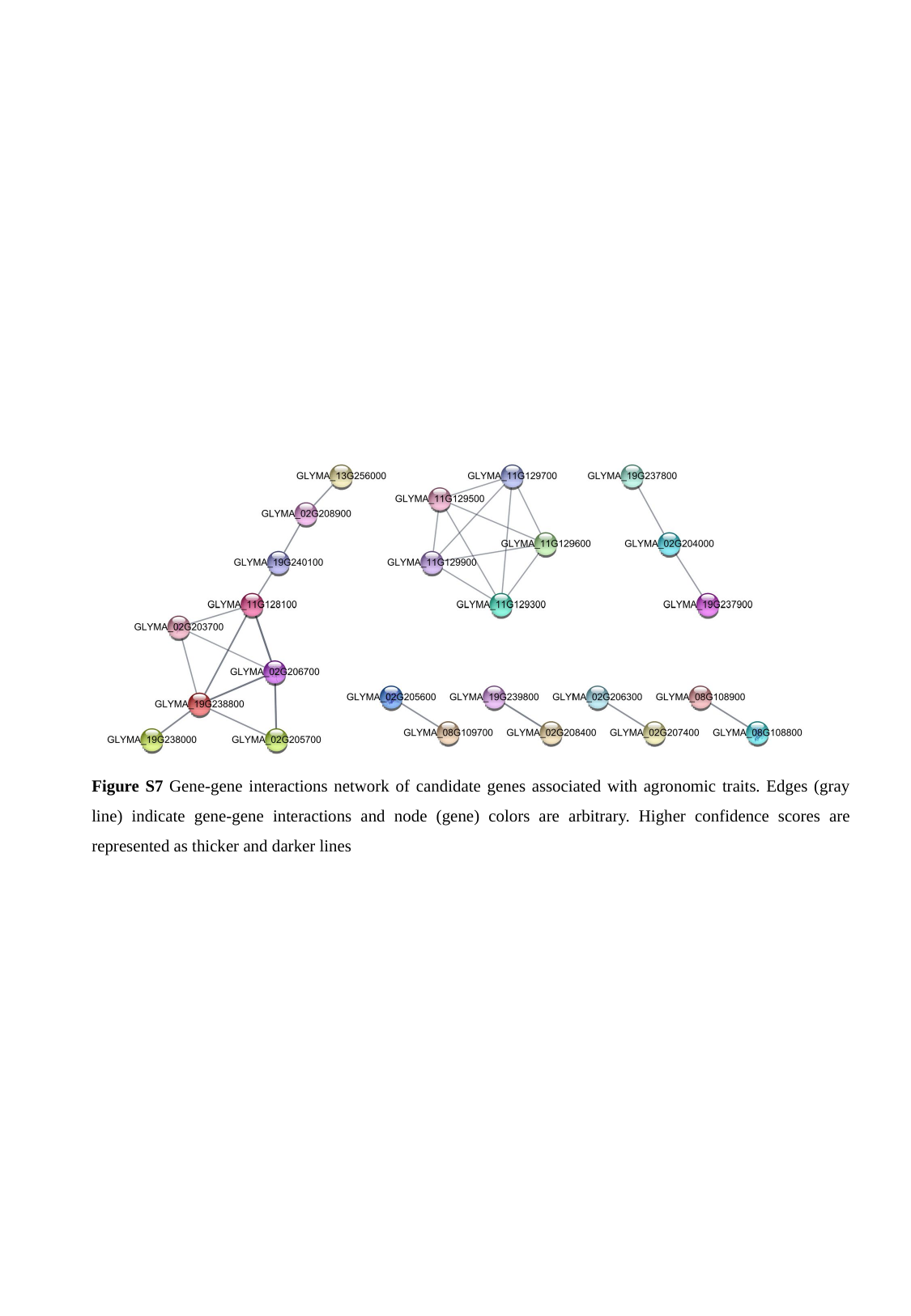

Figure S7 Gene-gene interactions network of candidate genes associated with agronomic traits. Edges (gray line) indicate gene-gene interactions and node (gene) colors are arbitrary. Higher confidence scores are represented as thicker and darker lines
